# Identification of Potential Distinguishing Markers for the Use of Cannabis-Based Medicines or Street Cannabis in Serum Samples

**DOI:** 10.3390/metabo11050316

**Published:** 2021-05-13

**Authors:** Anne Scheunemann, Katrin Elsner, Tanja Germerott, Sergiu Groppa, Cornelius Hess, Isabelle Miederer, Alicia Poplawski, Jörg Röhrich

**Affiliations:** 1 Institute of Legal Medicine, University Medical Center of the Johannes Gutenberg University Mainz, 55131 Mainz, Germany; elsner@uni-mainz.de (K.E.); germerott@uni-mainz.de (T.G.); hess@uni-mainz.de (C.H.); roehrich@uni-mainz.de (J.R.); 2 Department of Neurology, University Medical Center of the Johannes Gutenberg University Mainz, 55131 Mainz, Germany; segroppa@uni-mainz.de; 3 Department of Nuclear Medicine, University Medical Center of the Johannes Gutenberg University Mainz, 55131 Mainz, Germany; Isabelle.Miederer@unimedizin-mainz.de; 4 Institute of Medical Biostatistics, Epidemiology and Informatics, University Medical Center of the Johannes Gutenberg University Mainz, 55131 Mainz, Germany; alpoplaw@uni-mainz.de

**Keywords:** cannabinoids, Sativex, Dronabinol, medical cannabis, serum concentrations, LC-MS/MS, principal component analysis

## Abstract

Increasing prescription numbers of cannabis-based medicines raise the question of whether uptake of these medicines can be distinguished from recreational cannabis use. In this pilot study, serum cannabinoid profiles after use of cannabis-based medicines were investigated, in order to identify potential distinguishing markers. Serum samples after use of Sativex^®^, Dronabinol or medical cannabis were collected and analyzed for 18 different cannabinoids, using a validated liquid chromatography-tandem mass spectrometry (LC-MS/MS) method. Analytes included delta-9-tetrahydrocannabinol, 11-hydroxy-tetrahydrocannabinol, 11-nor-9-carboxy-tetrahydrocannabinol, cannabidiol, cannabinol, cannabigerol, cannabichromene, cannabicyclol, tetrahydrocannabivarin, cannabidivarin, tetrahydocannabinolic acid A, cannabidiolic acid, cannabinolic acid, cannabigerolic acid, cannabichromenic acid, cannabicyclolic acid, tetrahydrocannabivarinic acid and cannabidivarinic acid. Cannabinoid profiles of study samples were compared to profiles of street cannabis user samples via principal component analysis and Kruskal–Wallis test. Potential distinguishing markers for Dronabinol and Sativex^®^ intake were identified, including 11-hydroxy-tetrahydrocannabinol/delta-9-tetrahydrocannabinol ratios ≥1 and increased concentrations of 11-nor-9-carboxy-tetrahydrocannabinol, cannabidiol or cannabichromene. Larger quantities of minor cannabinoids suggested use of cannabis. Use of medical and street cannabis could not be distinguished, except for use of a cannabidiol-rich strain with higher cannabidiol/delta-9-tetrahydrocannabinol and cannabichromene/delta-9-tetrahydrocannabinol ratios. Findings of the study were used to classify forensic serum samples with self-reported use of cannabis-based medicines.

## 1. Introduction

For decades, *cannabis sativa L.* has been the most popular illicit drug worldwide, with a prevalence of 192 million in 2018 and rising numbers of users [1,2]. Psychoactive and neurotoxic properties of the plant’s main cannabinoid delta-9-tetrahydrocannabinol (THC), such as impairment of cognition, psychomotor function and impulse control, are especially problematic in road traffic [3,4]. In Germany, conducting a motor vehicle with a THC blood serum level above 1 ng/mL leads to a fine and suspension of the driver’s license.

Interest in therapeutic use of cannabinoids, not only THC, but also cannabidiol (CBD) or other, minor cannabinoids, has recently increased as well [5,6]. In Germany, cannabis-based medicines, such as Sativex^®^ or Dronabinol, as well as medical cannabis, can be prescribed for different indications and are reimbursable by health insurance funds since the amendment to the Narcotic Drugs Act in 2017. Sativex^®^ is an oromucosal spray which contains approximately equal amounts of THC and CBD as main ingredients [7]. Dronabinol, mainly prescribed as a 2.5% magistral preparation for oral application, contains mostly THC. Medical cannabis contains a complex mixture of many phytocannabinoids. Several THC-/CBD-predominant or mixed varieties are available [8].

Regarding participation in road traffic under influence of THC, intake of medicines is excluded from legal regulations. Thus, increasing prescription numbers of medical cannabis and cannabis-based medicines, about 150,000 in the first half of 2020 [9,10], raise the question of whether uptake of these medicines can be distinguished from recreational use of street cannabis. Minor cannabinoids such as tetrahydrocannabivarinic acid (THCVA) or tetrahydocannabinolic acid A (THCAA) have been suggested as markers for cannabis use [11,12], when cannabis-based medicines contain THC exclusively. However, low quantities of minor cannabinoids were previously observed in material samples of Sativex^®^ and Dronabinol [13]. It is not yet known, to which amount those minor cannabinoids are reflected in biological samples of users. Street and medical cannabis both derive from the same monospecific plant *Cannabis sativa L.* [14]. However, past studies observed that material samples of some medical cannabis varieties and street cannabis exhibited characteristic cannabinoid patterns [13,15], suggesting a differentiation in biological samples of users might be possible.

Regarding these findings, the aim of the here-presented multi-centered pilot study was to investigate serum cannabinoid concentrations after use of different cannabis-based medicines or medical cannabis in order to identify potential distinguishing markers from street cannabis use. Serum samples of patients treated with medical cannabis or cannabis-based medicines were collected, along with information about gender, age, body mass index (BMI), dosing regimen of the patients and time between medicine intake and sample collection. Serum samples were analyzed for 18 different cannabinoids, using a validated liquid chromatography-tandem mass spectrometry (LC-MS/MS) method [16]. Analytes included THC, CBD, cannabinol (CBN), cannabigerol (CBG), cannabichromene (CBC), cannabicyclol (CBL), tetrahydrocannabivarin (THCV) and cannabidivarin (CBDV) and their acidic precursors tetrahydocannabinolic acid A (THCAA), cannabidiolic acid (CBDA), cannabinolic acid (CBNA), cannabigerolic acid (CBGA), cannabichromenic acid (CBCA), cannabicyclolic acid (CBLA), tetrahydrocannabivarinic acid (THCVA) and cannabidivarinic acid (CBDVA) as well as the THC metabolites 11-hydroxy-tetrahydrocannabinol (THC-OH) and 11-nor-9-carboxy-tetrahydrocannabinol (THC-COOH). Subsequently, cannabinoid profiles of study samples were compared to profiles of street cannabis user samples via principal component analysis (PCA) and Kruskal–Wallis test. Potential distinguishing markers for Sativex^®^ and Dronabinol intake were identified and used to classify nine forensic serum samples with self-reported use of cannabis-based medicines.

## 2. Results and Discussion

### 2.1. Demographics and Therapy of Study Population

A total of 23 participants (13 female and 10 male), aged 23 to 78 with body mass indexes (BMIs) ranging from 15 to 55, were included in the study, and 56 serum samples in total were obtained from the collective. Three patients were treated with Sativex^®^ (*n* = 9 samples), two with both Sativex^®^ and Bedrocan^®^ (*n* = 6), five with one or more types of medical marihuana (Bediol^®^, Bedrocan^®^, Bedrolite^®^, Pedanios 22/1 or Bedrobinol^®^, *n* = 13), one participant reported use of Dr. Nice Rebound CBD capsules (*n* = 1), 10 participants were treated with 2.5% Dronabinol solution according to Neues Rezeptur-Formularium (NRF) 22.8 (*n* = 14) and two participants reported treatment with 2.5% Dronabinol solution NRF 22.8 and additional use of street cannabis (*n* = 6). Daily doses applied ranged from 4 to 39 drops of Dronabinol, 2 to 20 sprays of Sativex^®^ and 50 mg to 3 g of medical cannabis. Time between intake of the cannabis-based medicine and collection of the blood sample ranged from 33 min to approximately 5 d 15 h. An overview of demographics and the participants’ therapy including approximate doses of THC applied is provided in Supplementary Table S1.

### 2.2. Quantitative Study Sample Analysis

Within the study collective, THC was detected in 48 of 56 samples, ranging from approximately 0.048 (value below limit of quantification (LOQ)) to 49 ng/mL. THC-OH and THC-COOH concentrations ranged from approx. 0.20 to 26 ng/mL and approx. 1.0 to 29 ng/mL. Among minor cannabinoids, THCAA, CBN, CBC and THCVA were detected in most samples with maximum values ranging from 0.97 (CBN) to 9.3 ng/mL (THCAA). Other analytes, such as CBG or THCV, rarely occurred. CBDVA was detected in one sample only, and CBL and CBDV were not detected in any samples. Serum cannabinoid concentrations itemized by substance intake along with time intervals between last substance intake and blood sample collection are presented in Table 1.

#### 2.2.1. Sativex^®^

In the sub-collective of Sativex^®^ patients, time intervals between last intake and blood draw ranged from 33 min to 18 h 20 min. THC was detected in all samples. Serum concentrations ranged from approx. 0.15 to 4.6 ng/mL, with THC-OH levels of approx. 0.36 to 22 ng/mL and THC-COOH levels of 19 to 170 ng/mL. As expected, CBD occurred in all samples as well, with concentrations ranging from 0.23 to 6.1 ng/mL. Its acidic precursor CBDA was detected in 6 of 9 samples, ranging from approx. 0.0020 to 0.042 ng/mL. Among other minor cannabinoids, CBC exhibited the highest concentrations with approx. 0.11 to 1.1 ng/mL. THCAA, CBN and THCVA were ubiquitous in samples of Sativex^®^ patients but exhibited relatively low maximum values of 0.084 ng/mL (THCAA) and 0.044 ng/mL (CBN). THCVA concentrations lay below LOQ in all samples. The results lie in accordance with findings of a previous study regarding the phytocannabinoid profile of Sativex^®^ [13], in which, besides THC and CBD, it mainly exhibited CBC (0.2%). Interestingly, despite rather low quantities of other minor cannabinoids in the extract (THCAA 0.001%, CBN 0.003%, THCVA 0.000004%), those cannabinoids were detected in serum samples of users.

#### 2.2.2. Sativex^®^ and Medical Cannabis

Serum samples of Sativex^®^ patients with additional use of medical cannabis (Bedrocan^®^) exhibited similar cannabinoid patterns as those of Sativex^®^ patients. Concentrations for THC, THC-OH and THC-COOH ranged from approx. 0.10 to 1.4 ng/mL, 0.63 to 2.4 ng/mL and 2.1 to 56 ng/mL, respectively. THCAA, CBD, CBDA, CBN and CBC occurred in most samples. THCVA was ubiquitous and exhibited higher concentrations than in Sativex^®^ patients, with a maximum value 1.8 ng/mL. Time intervals between substance intake and blood sample collection were similar to those in the Sativex^®^ subgroup. However, the minimum was approximately three times as high with 1 h 45 min.

#### 2.2.3. Medical Cannabis

Samples of medical cannabis users exhibited the highest THC concentrations with a maximum of 49 ng/mL. THC-OH and THC-COOH levels ranged from approx. 0.27 to 26 ng/mL and 2.2 to 180 ng/mL, respectively. Compared to Sativex^®^ users, CBD occurred less frequently and mostly at concentrations <LOQ after use of the THC dominant strains Bedrocan^®^ or Bedrobinol^®^. Use of the CBD-rich variety Bediol^®^, however, resulted in a higher serum level of 2.0 ng/mL. Other minor cannabinoids, such as CBNA, CBG and THCV, occurred most frequently in medical cannabis user samples, compared to other subgroups. Additionally, the samples exhibited the highest concentrations for THCAA, CBN and THCVA within the whole collective, with maximum values of 9.3 ng/mL, 0.97 ng/mL and 3.2 ng/mL, respectively. Time intervals between intake of medical cannabis and blood draw varied greatly, ranging from 1 h 10 min to approximately 5 d 15 h.

#### 2.2.4. Dronabinol and Street Cannabis

Cannabinoid patterns of participants using Dronabinol and street cannabis were similar to those of medical cannabis users. THC concentrations ranged from 0.41 to 16 ng/mL, with THC-OH and THC-COOH levels of approx. 0.26 to 12 and 3 to 290 ng/mL. THCAA, CBN, CBGA and THCVA were detected in all, and CBG, CBC and CBCA in most samples. CBD and CBDA, however, were almost completely absent in this sub-collective. This is consistent with observed high popularity of THC-rich cannabis strains with very little CBD [1]. Time intervals between substance intake and blood sample collection were also similar to the medical cannabis user subgroup, ranging from 2 h 47 min to approximately 4.5 d.

#### 2.2.5. Dronabinol

Samples of Dronabinol users exhibited rather low THC concentrations of approx. 0.048 to 1.0 ng/mL, with THC-OH and THC-COOH levels ranging from approx. 0.20 to 2.7 ng/mL and approx. 1.0 to 29 ng/mL, respectively. As expected, minor cannabinoids were almost completely absent from the samples. In some cases, THCAA, CBN, CBGA and THCVA were detected, but concentrations mostly lay below LOQ. This is consistent with previous findings [13], in which the plant-derived Dronabinol extract exhibited mostly THC and very low concentrations of other minor cannabinoids. Time intervals between intake of Dronabinol and blood draw ranged from 1 h to approximately 24 h.

#### 2.2.6. Dr. Nice Rebound CBD Capsules

Although Dr. Nice Rebound CBD capsules contain hemp extract with respectable quantities of THC and CBC, according to the manufacturer [17], no THC, THC metabolites or CBC were detected after intake of the capsules. This can be explained by a rather low dose of only one capsule per day and a long-time interval of more than 14 h between intake and collection of the serum sample (see Supplementary Table S1). Besides CBD (0.24 ng/mL), the serum sample exhibited notable concentrations of many acidic minor cannabinoids, such as CBDA (0.37 ng/mL), CBGA (0.48 ng/mL), CBCA (0.18 ng/mL) or CBDVA (0.018 ng/mL) that were absent in most of the other sub-collectives. The absence in other subgroups can be explained by thermal degradation of acidic cannabinoids due to smoking or vaporizing cannabis, in contrast to oral application in this subgroup.

### 2.3. Comparison to Forensic Serum Samples and Identification of Potential Distinguishing Markers

**Figure 1:**
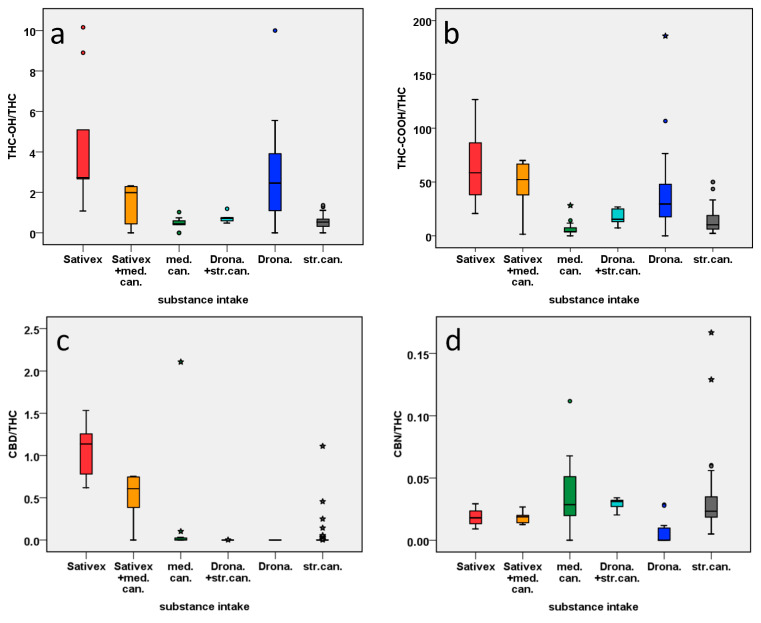

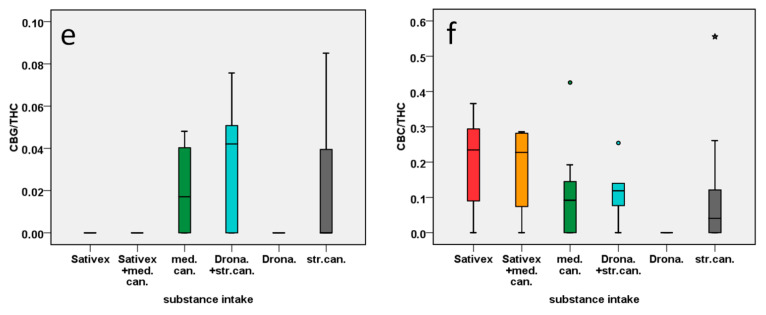
Boxplots of selected cannabinoids illustrate differences among subgroups. In Sativex^®^ user samples, standardized THC-OH (**a**), THC-COOH (**b**) and CBD (**c**) concentrations were higher than for medical cannabis (med.can.) or street cannabis (str.can) users. Dronabinol (Drona.) users also exhibited higher THC-OH and THC-COOH levels but fewer CBN (**d**) and CBC (**f**) than cannabis users. Cannabis user samples exhibited notably higher levels of CBG (**e**), compared to Sativex^®^ or Dronabinol users. * = Extreme outliers with values ≥3, * Interquartile Range (First Quartile–third Quartile).

In order to identify potential distinguishing markers for the intake of cannabis-based medicines or use of street cannabis, all study subgroups, except for the CBD capsule group with n = 1, were compared to 55 forensic serum samples of different street cannabis users, that had been analyzed in a previous study [16]. Serum cannabinoid concentrations of forensic case samples (in the following referred to as street cannabis subgroup) are presented in Supplementary Table S2. For better comparability of different doses of cannabis or cannabis-based medicine applied as well as different time intervals between application, cannabinoid concentrations were standardized on THC by dividing them by the respective THC concentration of the sample. Resultant cannabinoid ratios of all samples are listed in Supplementary Table S3. For eight samples, no ratios could be calculated, as THC serum concentrations lay below limit of detection (LOD). Those samples were excluded from further analysis. If other cannabinoid concentrations lay below LOD, 0 (zero) was substituted as the respective ratio. CBDV was not detected in any samples and was therefore eliminated from further analyses. Boxplots of selected cannabinoid ratios are presented in Figure 1.

#### 2.3.1. Statistical Analysis via Principal Component Analysis and Kruskal–Wallis Test 

**Figure 2:**
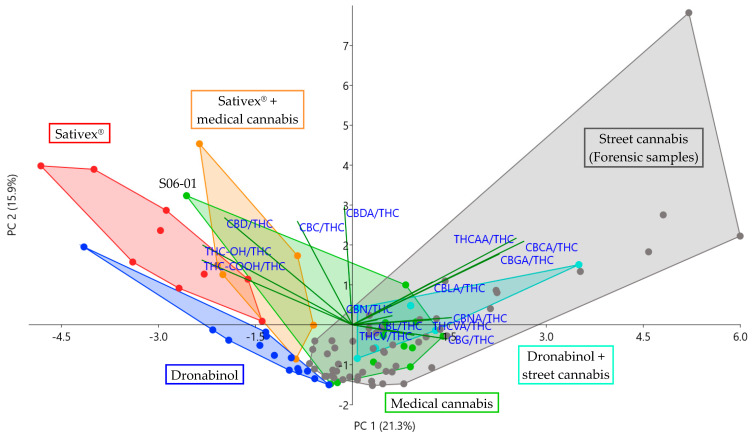
Sample subgroups separated in the PCA biplot of principal component (PC) 1 and PC 2 with loadings expressed as vectors. The orientation of a vector illustrates which PC Table 1. and the positive axis of PC 2. The further away a vector from the origin of a PC is, the higher the loading on the PC. Samples of Dronabinol and Sativex^®^ users are separated from street cannabis users, indicating different serum cannabinoid patterns. Samples from users of street cannabis, medical cannabis or Dronabinol and street cannabis are mostly plotted in the same area, indicating similar serum cannabinoid patterns, except for sample S06-01 from a user of the CBD-rich strain Bediol^®^.

A principal component analysis (PCA) of all cannabinoid ratios, standardized to z-scores as described above, was carried out. Samples F03 and F21 were identified as outliers, as they were plotted far away from the rest of the collective, distorting the biplot. For this reason, F03 and F21 were excluded. After exclusion, transformation into z-scores and PCA were repeated, and sufficient results were achieved. With a value of 0.650, the Kaiser–Meyer–Olkin measure of sampling adequacy was sufficient for performing a factor analysis [18,19]. Bartlett’s test of sphericity was significant (*p* < 0.001), indicating sufficiently large correlations between items for performing a PCA [19]. The biplot of PC 1 and 2 in Figure 2 shows how different subgroups were separated via PCA. Corresponding loading plots for PC 1 and 2 are displayed in Supplementary Figure S1.

Additionally, an analysis of variance was performed on all subgroups. As data were not normally distributed, when tested via Kolmogorov–Smirnov and Shapiro–Wilk test, comparison was carried out via Kruskal–Wallis test [20]. In view of the pilot character and small sample size of this study, both uncorrected *p* values and *p* values adjusted via Bonferroni–Holm method were used for interpretation of study results [21]. In addition, differences with uncorrected *p* values ≥ 0.05 were reported when observed in both PCA comparison and Kruskal–Wallis test. Differences and according effect sizes for selected pairwise comparisons of the subgroups are presented in Table 2.

#### 2.3.2. Sativex^®^ vs. Cannabis or Combination of Sativex^®^ and Cannabis

In the PCA biplot presented in Figure 2, Sativex^®^ samples were well separated from street cannabis and most medical cannabis user samples, the only exception being medical cannabis sample S06-01 after use of the CBD-rich strain Bediol^®^. PC 1 and PC 2 together covered more than 37% of the total variance among samples, indicating a relatively high degree of confidence. Located in the second quadrant in the biplot, Sativex^®^ user samples featured higher ratios of THC-OH/THC, THC-COOH/THC, CBD/THC and CBC/THC than cannabis-related subgroups, while ratios of other minor cannabinoids were rather low. These results are confirmed by boxplot comparisons in Figure 1. Figure 1a shows that THC-OH/THC ratios were >1, which lies in accordance with previous findings [22,23]; Nadulski et al. discovered that, due to an extensive first-pass metabolism, THC-COOH and THC-OH plasma concentrations were elevated after oral use of THC, compared to smoking, and the respective THC-OH/THC ratio was almost always >1 within the first 2 h after consumption. According to Karschner et al., oromucosal use of THC resulted in similar THC-OH/THC ratios as oral use. In comparison to the Sativex^®^ subgroup, and in accordance with [23] and [22], standardized THC-COOH and especially THC-OH concentrations of street or medical cannabis users were much lower, and THC-OH/THC ratios were almost always <1. Almost equal quantities of CBD and THC in Sativex^®^ were mirrored in serum samples as well, analog to previous findings in plasma and oral fluid [24,25]. CBD/THC ratios ranged from 0.6 to 1.5, as illustrated in Figure 1c. In comparison, ratios were much lower in cannabis user subgroups. The only exception was formed by Sativex^®^ users with additional intake of medical cannabis. This could be due to relatively low daily dosages of medical cannabis (see Supplementary Table S1) with hardly any influence on THC serum levels. Proportions of CBD and THC could be more similar to those of Sativex^®^ users, if increasingly popular CBD-rich cannabis or additional CBD is consumed [26]. However, inhalatory and oral uptake of THC could still be differentiated via THC-OH/THC ratio. Only low ratios of minor cannabinoids, such as THCAA/THC, THCVA/THC, CBGA/THC or CBCA/THC, observed in Sativex^®^ user samples are consistent with previous suggestions of minor cannabinoids as markers [11,12]. Consequently, despite possibly increased THC-OH/THC ratios, oral administration of street cannabis could still be distinguished due to increased levels of minor cannabinoids. Unless a CBD-rich strain is used, CBD/THC ratios should differ from those of Sativex^®^ users as well. Observations mentioned above were confirmed by the Kruskal–Wallis test, when comparing Sativex^®^ and street or medical cannabis users to each other. Most of the time, differences in ratios of THC-OH, THC-COOH, CBD and other minor cannabinoids were significant and had medium or strong effect sizes. Results of the Kruskal–Wallis test are presented in Table 2.

#### 2.3.3. Dronabinol vs. Cannabis or Combination of Dronabinol and Cannabis

Samples of Dronabinol users were separated from users of street or medical cannabis or street cannabis additional to Dronabinol in Figure 2. Mostly located on the negative axis of PC 1 and PC 2, the Dronabinol subgroup was characterized by an almost exclusive presence of THC, THC-OH and THC-COOH and very few other cannabinoids. As illustrated in Figure 1a, THC-OH/THC ratios were >1, mirroring oral intake of THC [22,23] in contrast to ratios mostly <1 after smoking or vaporizing medical or street cannabis. Whereas traces of minor cannabinoids such as THCAA or CBN occurred sporadically in Dronabinol user samples, larger concentrations could indicate an additional use of cannabis, as demonstrated for the Dronabinol user group with additional cannabis use in Figure 2. The findings are undermined by results of the Kruskal–Wallis test presented in Table 2: Significant differences with strong effect sizes were discovered for most analytes, including THC-OH/THC and THC-COOH/THC ratios, as well as minor cannabinoids, such as CBN, CBG or CBC. Differences of the latter are visualized in boxplots in Figure 1d–f.

#### 2.3.4. Medical Cannabis vs. Street Cannabis

Medical and street cannabis user samples mostly overlapped in the PCA plot in Figure 2, indicating similar cannabinoid serum patterns. A pairwise comparison via Kruskal–Wallis test (see Table 2) showed differences in THC-COOH/THC, CBD/THC, CBDA/THC and CBNA/THC ratios with medium effect sizes. Greater CBNA/THC and especially THC-COOH/THC ratios in street cannabis users (see Supplementary Table S3) could be explained by greater residual cannabinoid quantities after frequent use for recreational purposes, compared to medical cannabis patients with a fixed dosing regimen. Moreover, ranges for THC-COOH/THC in medical and street cannabis users partially overlapped in Figure 1b. The only striking difference between the subgroups was discovered after use of the CBD-rich strain Bediol^®^: Interestingly, sample S06-01 was well separated from both other medical and street cannabis user samples in the PCA plot. Besides CBD/THC, it also exhibited the highest CBC/THC ratio among cannabis user samples. Samples from a user of the CBD-rich strain Bedrolite^®^ were not separated from the rest of the subgroup. However, the dosage regimen was rather low for this participant, and Bedrolite^®^ was combined with the THC-rich variety Bedrocan^®^. The results suggest that use of CBD-rich medical cannabis strains might be distinguishable by different serum cannabinoid patterns. This observation lies in accordance with previous findings, in which material samples of medical cannabis strains exhibited characteristic cannabinoid patterns [13,15]. However, further studies with larger case numbers and users of more different medical cannabis strains are required to explore differentiability in serum samples. Moreover, fluctuations in cannabinoid profiles among different medical cannabis batches should be regarded [15,27,28].

### 2.4. Application to Forensic Case Samples

**Figure 3:**
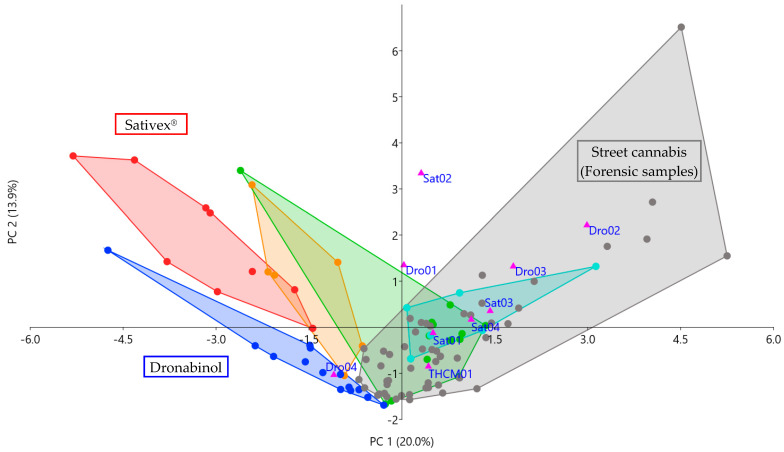
PCA scatter plot of principal component (PC) 1 and PC 2 shows that forensic samples with reported intake of cannabis-based medicines (pink triangles) are mostly plotted among street cannabis user samples (grey dots). Full color code is given in Figure 2. Loadings of PC 1 and PC 2 are given in supplementary Figure S2. Only one sample with self-reported Dronabinol intake (Dro04) was plotted among samples of Dronabinol users (blue dots), indicating similar serum cannabinoid patterns that could indeed result from Dronabinol use. All samples with self-reported use of Sativex^®^ (Sat01-Sat04) were plotted far from the Sativex^®^ user sub-collective (red dots) but rather among forensic samples, suggesting (additional) use of cannabis or cannabis-based products with notable quantities of minor cannabinoids.

Results of the presented study were used to analyze nine forensic case serum samples with self-reported intake of Sativex^®^ (n = 4, named Sat01 to Sat04), Dronabinol (n = 4, Dro01 to Dro04) and “THC medicine” (n = 1, THCM01). Cannabinoid concentrations of the samples were quantified as described above and are presented in Table 3. Cannabinoid concentrations standardized on THC are given in Supplementary Table S4. Standardized cannabinoid concentrations were analyzed in a PCA along with study and forensic samples (except for F03 and F21) described in 3.2. With a value of 0.606, the Kaiser–Meyer–Olkin measure of sampling adequacy was sufficient for this dataset, and Bartlett’s test of Sphericity was significant (*p* < 0.001) [18,19]. The resultant PCA scatter plot of all samples plotted on PC 1 and PC 2 is presented in Figure 3. Corresponding loading plots of PC 1 and PC 2 are displayed in Supplementary Figure S2.

Samples with reported Sativex^®^ intake exhibited different cannabinoid patterns than Sativex^®^ users from the study: Especially THC-OH/THC and CBD/THC ratios were much lower, ranging from 0.37 to 0.71 and 0.042 to 0.23, compared to ranges of 1.1 to 10 and 0.61 to 1.5 detected in Sativex^®^ user study samples. Minor cannabinoids, such as CBG, CBGA or THCV, on the other hand, occurred in higher quantities. As shown in Figure 3, cannabinoid patterns of forensic Sativex^®^ user samples were much more similar to medical or street cannabis users, suggesting use of cannabis or other cannabis-based products with notable quantities of minor cannabinoids. THC-OH/THC ratios <1 indicate inhalatory instead of oral application of THC. However, relatively high CBD and CBDA serum concentrations, compared to most street cannabis users, could be compatible with additional use of Sativex^®^.

Forensic samples with self-reported Dronabinol intake mostly exhibited different cannabinoid patterns as well: THC-OH/THC ratios ranged from 0.23 to 0.42 in samples Dro01 to Dro03, compared to 1.1 to 10 in Dronabinol user study samples, indicating THC had been inhaled instead of oral application. Larger quantities of other minor cannabinoids, such as CBG or CBC, suggest the use of cannabis or other cannabis-based products with notable quantities of minor cannabinoids. Accordingly, samples Dro01, Dro02 and Dro03 were plotted among cannabis user samples in Figure 3. Sample Dro04, however, exhibited a THC-OH/THC ratio of almost 1.6 and only low concentrations of other cannabinoids. It was plotted among Dronabinol user study samples, indicating similar serum cannabinoid patterns that could indeed result from Dronabinol intake.

Sample THCM01 after self-reported intake of “THC medicine” featured a THC-OH/THC ratio of 0.24 and low concentrations of minor cannabinoids. It was plotted among cannabis user samples in Figure 3, suggesting inhalative use of cannabis or cannabis-based products. 

### 2.5. Limitations

This pilot study suffers from some limitations. The information about intake of the medicines and additional use of cannabis products is based on self-reports by study participants only and, therefore, is of limited reliability. This also accounts for reported time between intake of cannabis-based medicine and blood draw as well as last dose of the medicine. However, measuring and reported data seemed mostly compatible, and cannabinoid patterns were overall consistent within the sub-collectives. For some samples, such as S10-04, relatively high THC concentrations were detected, suggesting that time between last dose and sample collection might have been shorter than reported. A much bigger sample size might be required to investigate possible individual differences in metabolism of cannabinoids. Limited transferability of study results to other individuals should be noted, for example, when BMIs of the subjects or time span between last intake and sample collection lie outside the here investigated ranges. Multiple sample collection from the same individual in this study could potentially distort subgroup results. However, in the PCA plot, multiple samples from one individual did not cluster, compared to single samples of the same subgroup, but were relatively wide-spread. This makes a notable bias for the subgroup due to clustering of samples in one spot less likely. Little is known about metabolism and detection windows of minor cannabinoids, and different time intervals between substance intake and sample collection cannot be ruled out as a cause for different (minor) cannabinoid concentrations among subgroups. However, average time intervals of Sativex^®^ and Dronabinol users were shorter than those of medical cannabis users (approx. 6.3 h and 8.5 h compared to 14.4 h). Consequently, only low cannabinoid amounts in the first two subgroups are more likely due to only low concentrations in the medicines themselves. Findings of this pilot study could be confirmed in a controlled study setting with larger case numbers and collection of blank samples from participants before intake of cannabis products.

## 3. Materials and Methods

### 3.1. Study Participants

Inclusion criteria for the study were a minimum age of 18 years, treatment with a cannabis-based medicine, such as Dronabinol, Sativex^®^ or medical marihuana, and capacity of discernment. Exclusion criteria were an age <18 years, severe metabolic disorders that could impair metabolization of the cannabis-based medicine, such as severe liver or kidney dysfunctions, and impaired capacity of discernment. All participants provided written informed consent before being admitted to the study.

### 3.2. Study Design

In the multi-centered pilot study, a blood sample was collected from the participants, every time they had a medical appointment at one of the study centers involved. For blood sampling, 9 mL S-Monovette^®^ neutral blood tubes (Sarstedt AG und Co., Nümbrecht, Germany) were used. Information about gender, age, body height and weight, cannabis-based medicine received, dosage regimen, further intake of cannabis or cannabis-based products in the last seven days and time between last application of the medicine and blood draw was collected in a questionnaire after every sample collection. The study was carried out over a period of approximately 12 months. Following study centers were involved:

Department of Neurology—Movement Disorder and Neurostimulation Outpatient Clinic of the University Medical Center of the Johannes Gutenberg University Mainz

Department of Anesthesiology of the University Medical Center of the Johannes Gutenberg University Mainz

Department of Internal Medicine III of the University Medical Center of the Johannes Gutenberg University Mainz

Pain and Palliative Center Rhine-Main in Wiesbaden

Medical practice Dr. Löwenstein in Mainz

The study was approved by the ethics committees of the State Medical Associations of Rhineland-Palatinate and Hessen (application number 2018-13337).

### 3.3. Chemicals and Reagents

Reference standards for THC-d3, THC-OH, THC-OH-d3, THC-COOH-d9, CBD, CBD-d3, CBDA, CBN-d3, CBNA, CBG, CBGA, CBC, CBCA, CBL, THCV, THCVA, CBDV and CBDVA were purchased from Cerilliant (Round Rock, TX, USA), CBG-d9 and CBC-d9 from Cayman Chemical Co. (Ann Arbor, MI, USA) via LGC Standards (Wesel, Germany), CBN, THC and THC-COOH from LGC Standards and THCAA from THC Pharm (Frankfurt a. M., Germany). All standards were certified reference material and had a purity of at least 99.0%, except for THC-OH-d3, THCAA, CBC, CBC-d9, CBCA, CBL, THCVA, CBDV and CBDVA (97.7%), THC-d3 (96.7%), and CBG-d9 (95.0%). All chemicals were obtained from Carl Roth (Karlsruhe, Germany), except for methanol (from Fisher Scientific (Loughborough, UK)) and acetonitrile (from AppliChem (Darmstadt, Germany)). Chemicals used in LC-MS/MS measurement processes were LC-MS grade. All other chemicals were HPLC grade, except for acetone and dichloromethane, which had a purity of 99.8% and 99.5%, respectively. Blank human serum used for calibration was obtained from voluntary healthy blood donors via blood donation service and was tested to be drug-free.

### 3.4. Sample Preparation

Serum samples were prepared as described previously [16]. 500 µL serum, 50 µL ISTD (consisting of 0.2 ng/µL of THC-COOH-d9 and 0.02 ng/µL of each THC-d3, THC-OH-d3, CBD-d3, CBC-d9, CBN-d3 and CBG-d9) and 1.5 mL acetonitrile were mixed. The supernatant was diluted with 6 mL phosphate buffer (0.1 M, pH 6). A fully automated solid phase extraction (SPE) was performed subsequently, using a Gilson Aspec GX 271 Workstation (Gilson International B.V. Germany, Limburg an der Lahn, Germany). After the SPE column (Bond Elut C18 column; 300 mg, 3 mL, Agilent, Santa Clara, CA, USA) was conditioned with 6 mL methanol and 2 mL water at a flow rate of 1 mL/min, the sample mixture was applied. The column was flushed with 4 mL water and 4 mL water/methanol (80:20 *v/v*) and subsequently rinsed with 1 mL 0.1 M acetic acid. After drying the column for 10 min, the cannabinoid compounds were eluted with 3 mL acetone/dichloromethane (50:50 *v/v*) [29]. The extract was evaporated to dryness at 40 °C and transferred into glass vials with 200 µL ethyl acetate. After evaporation at 30 °C, the samples were reconstituted in 50 µL of an acetonitrile/methanol/water mixture (3:3:2). For both evaporation steps, nitrogen was used.

### 3.5. LC-MS/MS Analysis

Samples were analyzed, using a previously described validated analysis method [16]. Analysis was performed with an UHPLC 1290 Infinity from Agilent Technologies (Waldbronn, Germany) and an Agilent Technologies 6490 Triple Quadrupole mass spectrometer, using a C18 column (ZORBAX Eclipse Plus C18 Rapid Resolution HD 2.1 × 100 mm 1.8-µ, Agilent) at 30 °C with 5.00 µL sample injection volume at a flow rate of 0.6 mL/min. Mobile phase A was 5 mM ammonium formate in water, mobile phase B consisted of 0.1% (*v/v*) formic acid in acetonitrile. Analytes were separated using the following elution: 65% mobile phase B for the first 4.5 min, changed to 80% B and increased to 80.9% B at 7.50 min, changed to 100% B till 9.50 min. The column was re-equilibrated with 65% B for 3.5 min. Total run time was 13 min. Analytes were measured using electrospray ionization (ESI) in positive and negative reaction mode with a gas temperature of 250 °C, gas flow of 15 L/min, nebulizer pressure of 20 psi, sheath gas heater temperature of 400 °C, sheath gas flow of 12 L/min, capillary voltage of 4000 V and a nozzle voltage of 1000 V. For evaluation of the data, the Agilent Mass Hunter software B 09.00 was used. Analytes were calibrated in blank human serum.

### 3.6. Data Analysis

Data were analyzed and illustrated in boxplots, using Microsoft Office Excel 2010 (Microsoft Corp., Redmond, WA, USA) and IBM SPSS^®^ Statistics 23 (International Business Machines Corporation (IBM), Armonk, NY, USA).

In order to identify cannabinoid patterns and possible distinguishing markers among samples, cannabinoid concentrations were analyzed via principal component analysis (PCA), using SPSS^®^ and PAST software (version 4.02) for scientific data analysis (Øyvind Hammer, Natural History Museum, University of Oslo, Norway) [30,31]. PCA is a mathematical tool for dimension reduction and visualization of patterns, i.e., similarities and differences, in high-dimensional datasets: Multidimensional data can be displayed in a clear 2- or 3-dimensional coordinate system, even if the dataset itself comprises much more than two or three variables. Therefore, the original observations are projected onto principal components (PC), which are linear combinations of the original variables, and along which the variation of the data is maximal [32]. Positions of the observations on the PCs are called PC scores and are illustrated in PCA scatter plots. Correlations between original variables and PCs are called loadings and are described in loading plots [33]. In a biplot, PC scores and loadings (shown as vectors) can be visualized simultaneously. When clustering in similar areas, items should exhibit similar properties, while separated items can be expected to differ with regard to the respective vector. Often, datasets with different ranges of variances are standardized before analysis [34]. Otherwise, variables with large variances could dominate the PCs, and potentially important effects of variables with small ranges would not be detected. A common standardization method is to transform data into z-scores. Therefore, for every sample i, the difference of a value x and the respective arithmetic mean $\overset{\text{¯}}{x}$ of a variable j is divided by the respective standard deviation s. The resultant equation is $z_{\mathrm{ij}}=\frac{\left(x_{\mathrm{ij}}-{\;\overset{\text{¯}}{x}}_{j}\right)}{s_{j}}$ [34]. For more information, you are referred to the according literature [34,35,36]. In this study, two PCA were carried out: One regarded study and forensic samples of street cannabis users. A second analysis regarded study samples, forensic samples of street cannabis users and forensic samples with self-reported intake of cannabis-based medicines. Suitability of the datasets for PCA were tested via Kaiser-Meyer-Olkin measure and Barlett’s test [18,19]. Respective datasets were normalized to z-scores as described above [34]. Results were plotted in variance-covariance matrices, and the first two PCs were visualized in a biplot or scatter plot, respectively. Loading plots of the PCs were provided as supplementary data. For more information on the software, you are referred to the PAST manual [37].

Additionally, study subgroups were compared via Kruskal–Wallis test. For every pairwise comparison, resultant effect sizes r were interpreted according to Cohen [38]. Family-wise error rates were controlled via Bonferroni–Holm method [39,40].

## 4. Conclusions

In this pilot study, 18 different cannabinoids were quantified in serum samples after use of or a combination of Sativex^®^, Dronabinol, different medical cannabis varieties or street cannabis, using a validated LC-MS/MS method. Resultant cannabinoid serum patterns were compared among the subgroups, and potential differentiating markers for use of Sativex^®^ or Dronabinol in contrast to cannabis were identified. Markers for Dronabinol or Sativex^®^ included THC-OH/THC ratios ≥1 and increased levels of THC-COOH, CBD or CBC, whereas large quantities of other minor cannabinoids, such as THCAA, CBGA or CBCA, suggested cannabis use. Use of THC-rich medical and street cannabis could not be distinguished. However, one user sample of a CBD-rich medical cannabis variety was separated from other cannabis samples due to higher CBD/THC and CBC/THC levels, suggesting certain strains might be distinguishable. Further studies with larger case numbers and more different medical cannabis varieties could confirm this observation. Results of this study were used to test nine forensic case serum samples with self-reported intake of cannabis-based medicines. One sample exhibited cannabinoid serum patterns matching the self-reported medicine, indicating that Dronabinol could indeed have been used. For all other samples, cannabinoid serum patterns were similar to those of cannabis users, suggesting an (additional) intake of cannabis.

## Figures and Tables

**Table 1:** Cannabinoid serum concentrations of the study collective in [ng/mL].

Sample	Substance Intake	Time between Last Intake and Blood Draw	THC	THC-OH	THC-COOH	THCAA	CBD	CBDA	CBN	CBNA	CBG	CBGA	CBC	CBCA	CBL	CBLA	THCV	THCVA	CBDV	CBDVA
S01-01	Sativex	2 h 25 min	0.34	0.89	26	(0.016) *	0.36	nd	0.0085	nd	nd	nd	(0.14)	nd	nd	nd	nd	(0.023)	nd	nd
S01-02	Sativex	18 h 20 min	0.22	0.62	19	(0.012)	0.25	nd	(0.0032)	nd	nd	nd	nd	nd	nd	nd	nd	(0.019)	nd	nd
S01-03	Sativex	7 h 15 min	(0.15)	(0.36)	19	(0.0055)	0.23	nd	(0.0016)	nd	nd	nd	nd	nd	nd	nd	nd	(0.021)	nd	nd
S02-01	Sativex	9 h	1.1	1.2	23	0.028	0.75	(0.0020)	0.020	nd	nd	nd	(0.11)	nd	nd	nd	nd	(0.023)	nd	nd
S02-02	Sativex	16 h 15 min	1.3	4.5	54	0.047	1.0	(0.0028)	0.030	nd	nd	nd	0.29	nd	nd	nd	nd	(0.021)	nd	nd
S03-01	Sativex	50 min	0.82	7.3	48	0.056	1.0	0.010	0.024	nd	nd	nd	0.28	nd	nd	nd	nd	(0.015)	nd	nd
S03-02	Sativex	33 min	0.63	6.4	72	0.032	0.77	0.0054	0.016	nd	nd	nd	(0.16)	nd	nd	nd	nd	(0.017)	nd	nd
S03-03	Sativex	1 h 24 min	4.6	12	100	0.084	2.8	0.041	0.044	nd	nd	nd	0.82	(0.030)	nd	nd	nd	(0.019)	nd	nd
S03-04	Sativex	1 h 5 min	4.4	22	170	0.034	6.1	0.042	0.040	(0.0066)	nd	(0.011)	1.1	0.032	nd	nd	nd	(0.024)	nd	nd
S04-01	Sativex+ Bedrocan	10 h 44 min (Sativex)	1.4	0.63	2.1	nd	0.52	0.013	0.017	nd	nd	nd	(0.10)	(0.011)	nd	nd	nd	(0.024)	nd	nd
S05-01	Sativex+ Bedrocan	13 h 35 min (Sativex)	(0.10)	nd	6.8	(0.0062)	nd	nd	(0.0022)	nd	nd	nd	nd	nd	nd	nd	nd	(0.034)	nd	nd
S05-02	Sativex+ Bedrocan	2 h 15 min (Sativex)	0.43	1.0	22	(0.017)	0.26	0.0042	(0.0078)	nd	nd	nd	(0.10)	nd	nd	nd	nd	(0.032)	nd	nd
S05-03	Sativex+ Bedrocan	1 h 45 min (Sativex)	0.45	0.88	30	0.040	0.34	0.024	0.012	nd	nd	nd	(0.12)	nd	nd	nd	nd	0.047	nd	nd
S05-04	Sativex+ Bedrocan	3 h 30 min (Sativex)	1.1	2.4	56	0.036	0.64	0.0071	0.020	nd	nd	nd	0.28	(0.015)	nd	nd	nd	1.8	nd	nd
S05-05	Sativex+ Bedrocan	2 h 10 min (Sativex)	0.71	1.4	27	0.040	0.53	(0.0034)	0.010	nd	nd	nd	(0.18)	nd	nd	nd	nd	(0.029)	nd	nd
S06-01	Bediol	3 h 56 min	0.94	0.72	7.2	(0.013)	2.0	0.0054	0.048	nd	nd	nd	0.45	nd	nd	nd	nd	0.18	nd	nd
S07-01	Bedrocan	1 h 10 min	49	22	170	9.3	0.18	0.0088	0.97	0.022	2.4	1.1	4.5	0.58	nd	0.022	0.36	3.2	nd	nd
S07-02	Bedrocan	1 h 30 min	32	17	170	3.7	(0.071)	(0.0031)	0.48	(0.013)	1.5	0.54	3.0	0.21	nd	(0.0096)	0.23	2.7	nd	nd
S07-03	Bedrocan	2 h 30 min	43	26	150	1.9	(0.080)	(0.0035)	0.92	0.030	1.7	0.74	4.2	0.36	nd	0.014	0.25	0.91	nd	nd
S07-04	Bedrocan	1 h 10 min	48	21	180	1.6	(0.045)	0.0053	0.84	0.026	2.1	0.34	4.4	0.12	nd	(0.0071)	(0.17)	1.5	nd	nd
S08-01	Bedrolite+ Pedanios	10 h 10 min (Pedanios 22/1)	nd	nd	nd	nd	nd	nd	nd	nd	nd	nd	nd	nd	nd	nd	nd	(0.013)	nd	nd
S08-02	Bedrolite+ Bedrocan	9 h (Bedrolite)	nd	nd	nd	(0.011)	nd	nd	nd	(0.011)	nd	nd	nd	nd	nd	nd	nd	(0.017)	nd	nd
S08-03	Bedrolite+ Bedrocan	2 h 30 min (Bedrolite)	nd	nd	nd	(0.0094)	nd	nd	nd	nd	nd	nd	nd	nd	nd	nd	nd	(0.013)	nd	nd
S08-04	Bedrolite+ Bedrocan	2 h 30 min (Bedrocan)	(0.084)	nd	nd	(0.0049)	nd	nd	nd	nd	nd	nd	nd	nd	nd	nd	nd	nd	nd	nd
S09-01	Bedrocan	15 h 26 min	0.39	0.42	11	(0.0093)	(0.036)	0.0063	0.018	nd	nd	0.022	nd	nd	nd	nd	nd	0.37	nd	nd
S09-02	Bedrocan	10 h 55 min	0.42	(0.27)	6.4	0.047	nd	(0.0020)	0.012	nd	nd	0.024	nd	nd	nd	nd	nd	0.23	nd	nd
S09-03	Bedrocan	1 d 14 h 50 min	(0.17)	nd	2.2	(0.019)	nd	nd	0.019	nd	nd	nd	nd	nd	nd	nd	nd	0.27	nd	nd
S10-01	Bedrobinol	4 h 50 min	0.20	nd	nd	(0.012)	nd	nd	(0.0036)	nd	nd	nd	nd	nd	nd	nd	nd	(0.0087)	nd	nd
S10-02	Bedrobinol	3 h 20 min	3.7	1.5	14	0.88	(0.087)	nd	0.25	nd	0.090	0.033	0.64	(0.029)	nd	nd	nd	0.21	nd	nd
S10-03	Bedrobinol	1 h 55 min	4.7	2.0	20	1.3	(0.076)	nd	0.18	nd	(0.079)	0.054	0.85	0.055	nd	nd	nd	0.56	nd	nd
S10-04	Bedrobinol	5 d 14 h 40 min	2.1	1.1	8.4	0.45	(0.073)	nd	0.12	nd	(0.046)	(0.011)	0.27	(0.023)	nd	nd	nd	0.28	nd	nd
S11-01	Dronabinol+ Street cannabis	6 h 34 min (Dronabinol)	0.84	1.0	21	(0.016)	nd	nd	0.027	nd	nd	(0.0082)	(0.15)	nd	nd	(0.0021)	nd	0.29	nd	nd
S11-02	Dronabinol+ Street cannabis	8 h 19 min (Dronabinol)	1.4	1.0	21	0.17	nd	nd	0.029	(0.0049)	(0.057)	0.024	(0.18)	(0.013)	nd	nd	nd	2.3	nd	nd
S11-03	Dronabinol+ Street cannabis	approx. 4,5 d (Dronabinol)	0.41	(0.26)	2.5	0.11	nd	nd	0.014	(0.0045)	nd	(0.012)	nd	(0.011)	nd	(0.0024)	nd	0.31	nd	nd
S11-04	Dronabinol+ Street cannabis	12 h 50 min (Joint)	1.2	0.69	19	0.11	nd	nd	0.032	nd	(0.056)	(0.015)	0.27	(0.010)	nd	(0.0023)	nd	0.40	nd	nd
S12-01	Dronabinol+ Street cannabis	3 h (Dronabinol)	11	5.3	290	0.66	nd	nd	0.35	nd	0.83	0.14	1.3	(0.025)	nd	nd	nd	0.78	nd	nd
S12-02	Dronabinol+ Street cannabis	2 h 47 min (Dronabinol)	16	12	210	0.78	(0.044)	nd	0.47	0.020	0.66	0.11	1.2	0.032	nd	nd	nd	1.7	nd	nd
S13-01	Dronabinol	7 h 20 min	0.48	1.9	23	(0.0005)	nd	nd	nd	nd	nd	(0.0091)	nd	nd	nd	nd	nd	nd	nd	nd
S14-01	Dronabinol	1 h 20 min	0.82	0.93	16	(0.0004)	nd	nd	(0.0076)	nd	nd	nd	nd	nd	nd	nd	nd	0.18	nd	nd
S15-01	Dronabinol	1 h 40 min	(0.17)	0.53	13	nd	nd	nd	nd	nd	nd	nd	nd	nd	nd	nd	nd	nd	nd	nd
S15-02	Dronabinol	3 h 20 min	1.0	2.0	29	nd	nd	nd	(0.0024)	nd	nd	(0.0085)	nd	nd	nd	nd	nd	nd	nd	nd
S16-01	Dronabinol	23 h 40 min	(0.15)	(0.20)	2.4	(0.0025)	nd	nd	nd	nd	nd	nd	nd	nd	nd	nd	nd	nd	nd	nd
S16-02	Dronabinol	24 h 30 min	(0.048)	nd	2.4	nd	nd	nd	nd	nd	nd	nd	nd	nd	nd	nd	nd	nd	nd	nd
S16-03	Dronabinol	3 h 17 min	0.23	0.65	6.6	(0.017)	nd	nd	nd	nd	nd	nd	nd	nd	nd	nd	nd	nd	nd	nd
S17-01	Dronabinol	6 h 20 min	0.25	(0.27)	6.3	(0.015)	nd	nd	nd	nd	nd	nd	nd	nd	nd	nd	nd	nd	nd	nd
S17-02	Dronabinol	6 h 45 min	(0.067)	0.71	13	(0.017)	nd	nd	(0.0022)	nd	nd	nd	nd	nd	nd	nd	nd	nd	nd	nd
S17-03	Dronabinol	15 h 6 min	nd	nd	nd	nd	nd	nd	nd	nd	nd	nd	nd	nd	nd	nd	nd	nd	nd	nd
S17-04	Dronabinol	15 h 30 min	nd	nd	(1.0)	(0.0021)	nd	nd	nd	nd	nd	nd	nd	nd	nd	nd	nd	nd	nd	nd
S17-05	Dronabinol	15 h 25 min	nd	nd	nd	nd	nd	nd	nd	nd	nd	nd	nd	nd	nd	nd	nd	nd	nd	nd
S18-01	Dronabinol	2 h 5 min	(0.12)	nd	nd	(0.0044)	nd	nd	nd	nd	nd	nd	nd	nd	nd	nd	nd	nd	nd	nd
S19-01	Dronabinol	1h	(0.18)	1.0	2.8	0.031	nd	nd	(0.0053)	nd	nd	nd	nd	nd	nd	nd	nd	nd	nd	nd
S19-02	Dronabinol	Missing data	nd	nd	(1.6)	(0.012)	nd	nd	nd	nd	nd	nd	nd	nd	nd	nd	nd	nd	nd	nd
S20-01	Dronabinol	1 to 1,5 h	(0.17)	nd	3.4	nd	nd	nd	(0.0021)	nd	nd	nd	nd	nd	nd	nd	nd	nd	nd	nd
S21-01	Dronabinol	3 h 24 min	0.69	2.7	28	(0.0080)	nd	nd	(0.0046)	(0.0054)	nd	nd	nd	nd	nd	nd	nd	nd	nd	nd
S22-01	Dronabinol	13 h 54 min	(0.15)	0.46	16	nd	nd	nd	nd	nd	nd	nd	nd	nd	nd	nd	nd	(0.032)	nd	nd
S23-01	CBD capsules	14 h 15 min	nd	nd	nd	0.10	0.24	0.37	(0.0043)	(0.0062)	nd	0.48	nd	0.18	nd	(0.0041)	nd	0.42	nd	0.018

* Measured values below limit of quantification (LOQ) are reported as approximate values in parentheses, nd = not detected/below limit of detection (LOD).

**Table 2:** Results of the Kruskal–Wallis test for selected pairwise comparisons.

Pairwise Comparison	$\frac{THC-OH}{THC}$ (χ^2^ = 36.0, *p* = 0.000)	$\frac{THC-COOH}{THC}$ (χ^2^ = 40.2, *p* = 0.000)	$\frac{THCAA}{THC}$ (χ^2^ = 23.1, *p* = 0.000)	$\frac{CBD}{THC}$ (χ^2^ = 51.3, *p* = 0.000)	$\frac{CBDA}{THC}$ (χ^2^ = 36.5, *p* = 0.000)	$\frac{CBN}{THC}$ (χ^2^ = 30.3, *p* = 0.000)	$\frac{CBNA}{THC}$ (χ^2^ = 38.6, *p* = 0.000)	$\frac{CBG}{THC}$ (χ^2^ = 21.6, *p* = 0.0010)	$\frac{CBGA}{THC}$ (χ^2^ = 23.2, *p* = 0.000)	$\frac{CBC}{THC}$ (χ^2^ = 26.1, *p* = 0.000)	$\frac{CBCA}{THC}$ (χ^2^ = 14.3, *p* = 0.014)	$\frac{CBL}{THC}$ (χ^2^ = 1.76, *p* = 0.88)	$\frac{CBLA}{THC}$ (χ^2^ = 14.5, *p* = 0.013)	$\frac{THCV}{THC}$ (χ^2^ = 9.44, *p* = 0.093)	$\frac{THCVA}{THC}$ (χ^2^ = 36.8, *p* = 0.000)	$\frac{CBDVA}{THC}$ (χ^2^ = 0.873, *p* = 0.97)
**Sativex** vs. **Street Cannabis**	*z* = 4.82 *p* = 0 (0)	*z* = 4.17 *p* = 0 (0)	*z* = −2.79 *p* = 0.0050 (0.070)	*z* = 5.68 *p* = 0 (0)	*z* = 3.49 *p* = 0 (0)		*z* = −3.63 *p* = 0 (0)	*z* = −2.48 *p* = 0.013 (0.13)	*z* = −2.53 *p* = 0.011 (0.090)	z = 2.61 *p* = 0.0090 (0.090)					*z* = −3.22 *p* = 0.0010 (0.013)	
**Sativex** vs. **Medical Cannabis**	*z* = 4.35 *p* = 0 (0)	*z* = 4.95 *p* = 0 (0)	*z* = −1.84 *p* = 0.067 (0.67)	*z* = 3.01 *p* = 0.0030 (0.027)		*z* = −1.88 *p* = 0.060 (0.60)		*z* = −2.36 *p* = 0.018 (0.16)	*z* = −2.70 *p* = 0.0070 (0.084)				*z* = −1.83 *p* = 0.067 (0.64)		*z* = −1.79 *p* = 0.074 (0.67)	
**Sativex** vs. **Sativex + Medical Cannabis**	*z* = 1.85 *p* = 0.064 (0.64)															
**Dronabinol** vs. **Street Cannabis**	*z* = 3.60 *p* = 0 (0)	*z* = 3.29 *p* = 0.0010 (0.011)	*z* = −4.09 *p* = 0 (0)			*z* = −4.74 *p* = 0 (0)	*z* = −4.32 *p* = 0 (0)	*z* = −2.98 *p* = 0.0030 (0.042)	*z* = −2.62 *p* = 0.0090 (0.090)	*z* = −2.91 *p* = 0.0040 (0.048)	*z* =−3.02 *p* = 0.0030 (0.045)				*z* = −5.20 *p* = 0 (0)	
**Dronabinol** vs. **Medical Cannabis**	*z* = −3.20 *p* = 0.0010 (0.012)	*z* = -4.23 *p* = 0 (0)	*z* = 2.65 *p* = 0.0080 (0.10)	*z* = 3.05 *p* = 0.0020 (0.020)	*z* = 2.90 *p* = 0.0040 (0.036)	*z* = 4.15 *p* = 0 (0)		*z* = 2.66 *p* = 0.0080 (0.096)	*z* = 2.70 *p* = 0.0070 (0.084)	*z* = 2.92 *p* = 0.0040 (0.048)	*z* = 2.55 *p* = 0.011 (0.14)		*z* = 2.06 *p* = 0.039 (0.43)		*z* = 3.05 *p* = 0.0020 (0.024)	
**Dronabinol** vs. **Dronabinol + Street Cannabis**			*z* = 1.96 *p* = 0.050 (0.58)			*z* = 3.79 *p* = 0 (0)	*z* = 1.78 *p* = 0.075 (0.83)	*z* = 3.10 *p* = 0.0020 (0.030)	*z* = 3.06 *p* = 0.0020 (0.030)	*z* = 3.03 *p* = 0.0020 (0.026)	*z* = 2.99 *p* = 0.0030 (0.045)		*z* = 3.16 *p* = 0.0020 (0.030)		*z* = 4.10 *p* = 0 (0)	
**Street Cannabis** vs. **Medical Cannabis**		*z* = −2.10 *p* = 0.036 (0.29)		*z* = 2.39 *p* = 0.017 (0.12)	*z* = 2.58 *p* = 0.010 (0.080)		*z* = −3.64 *p* = 0 (0)									

Significant differences between subgroups were discovered with the Kruskal–Wallis test. For every variable, chi-square (χ^2^) and *p* values are given. Variables with insignificant *p* values ≥ 0.05 were shaded grey. For every pairwise comparison, z-scores, uncorrected *p* values and Bonferroni–Holm corrected *p* values (in parentheses) are reported. Cells were shaded yellow for medium effect sizes and red for large effect sizes. Effect sizes were reported for uncorrected *p* values ≥ 0.05, when differences were observed in the study as well (marked in lighter shades of yellow and red). The comparison of Sativex^®^ user samples to medical or street cannabis user samples showed especially large differences in THC-OH/THC, THC-COOH/THC and CBD/THC ratios. Samples of Dronabinol users differed greatly from cannabis users regarding almost all minor cannabinoids.

**Table 3:** Cannabinoid serum concentrations of forensic case serum samples with reported intake of cannabis-based medicines in [ng/mL].

Sample	Substance Intake	THC	THC-OH	THC-COOH	THCAA	CBD	CBDA	CBN	CBNA	CBG	CBGA	CBC	CBCA	CBL	CBLA	THCV	THCVA	CBDV	CBDVA
Sat01	Sativex	2.9	1.3	40	0.14	0.39	0.0043	0.12	nd	0.11	(0.015) *	0.42	nd	nd	nd	(0.048)	0.34	nd	nd
Sat02	Sativex	6.5	3.3	130	1.7	1.5	0.61	0.16	0.022	0.22	0.054	0.84	0.099	nd	nd	(0.026)	0.92	nd	nd
Sat03	Sativex	3.8	2.7	53	1.2	0.16	0.014	0.18	0.018	0.16	0.047	0.46	(0.024)	nd	nd	(0.080)	0.60	nd	nd
Sat04	Sativex	12	4.4	56	0.27	1.4	(0.0022)	2.0	0.050	0.46	0.027	1.8	(0.014)	nd	nd	(0.18)	0.36	nd	nd
Dro01	Dronabinol	0.96	0.4	30	0.050	0.31	nd	0.12	(0.011)	(0.048)	nd	0.36	nd	nd	nd	nd	0.29	nd	nd
Dro02	Dronabinol	1.3	(0.3)	23	0.046	nd	nd	0.053	nd	0.12	nd	0.26	0.16	nd	nd	nd	0.26	nd	nd
Dro03	Dronabinol	5.9	2.4	100	2.6	(0.063)	nd	0.19	0.022	0.35	0.19	1.7	0.039	nd	nd	(0.067)	2.9	nd	nd
Dro04	Dronabinol	(0.19)	(0.3)	8.6	(0.016)	nd	nd	(0.0015)	nd	nd	nd	nd	nd	nd	nd	nd	(0.031)	nd	nd
THCM01	THC medicine	2.1	0.5	9.8	0.12	(0.086)	nd	0.045	(0.014)	0.078	nd	(0.19)	(0.0091)	nd	nd	nd	0.87	nd	nd

* Measured values <LOQ are reported as approximate values in parentheses, nd = not detected/<LOD.

## Data Availability

The data presented in this study are available in Table 1, Table 2 and Table 3 and Supplementary Electronic Material.

## Supplementary Materials

The following are available online at https://www.mdpi.com/article/10.3390/metabo11050316/s1, Figure S1: Loading plot of principal component (PC) 1 and PC 2 for PCA of study and forensic samples, Figure S2: Loading plot of principal component (PC) 1 and PC 2 for PCA of study samples, forensic samples of street cannabis users and forensic samples with reported intake of cannabis-based medicines, Table S1: Demographic information on the study collective, Table S2: Cannabinoid concentrations of forensic samples analyzed in a previous study, Table S3: Cannabinoid concentrations of study and forensic samples standardized on THC, Table S4: Cannabinoid serum concentrations of forensic case samples with reported intake of cannabis-based medicines standardized on THC. References [41,42,43] are cited in the Supplementary Material.
