# Supplementary material for: Identification of Potential Distinguishing Markers for the Use of Cannabis-Based Medicines or Street Cannabis in Serum Samples
Source: Metabolites. 2021 May 13;11(5):316. doi: 10.3390/metabo11050316 (PMC8153355; doi:10.3390/metabo11050316)
Supplement: Supplementary file 1 [file metabolites-11-00316-s001.zip › metabolites-1212129-supplementary.pdf]

metabolites

MDPI

Supplementary Material

Identification of potential distinguishing markers for the use of cannabis-based medicines or street cannabis in serum samples

Anne Scheunemann [1,*], Katrin Elsner [1], Tanja Germerott [1], Sergiu Groppa [2], Cornelius Hess [1], Isabelle Miederer [3], Alicia Poplawski [4] and Jörg Röhrich [1]

[1] Institute of Legal Medicine, University Medical Center of the Johannes Gutenberg University Mainz, 55131 Mainz, Germany
[2] Department of Neurology, University Medical Center of the Johannes Gutenberg University Mainz, 55131 Mainz, Germany
[3] Department of Nuclear Medicine, University Medical Center of the Johannes Gutenberg University Mainz, 55131 Mainz, Germany
[4] Institute of Medical Biostatistics, Epidemiology and Informatics, University Medical Center of the Johannes Gutenberg University Mainz, 55131 Mainz, Germany
* Correspondence: anne.scheunemann@uni-mainz.de

Table S1. Demographic information on the study collective

Sample	Gender	Age	BMI*	Substance intake	Application	Dosing regimen per day	Approx. daily dose of THC [mg]***	Last dose before blood draw (respective THC quantity)	Time between last intake and blood draw
S01-01	f*	61	24	Sativex	om*	8 sprays per day	21.6 mg	M.D.*	2h 25min
S01-02	f	62	23	Sativex	om	3 - 0 - 3 sprays**	16.2 mg	3 sprays (8.1 mg THC)	18h 20min
S01-03	f	62	23	Sativex	om	2 - 0 - 3 sprays	13.5 mg	2 sprays (5.4 mg THC)	7h 15min
S02-01	m*	54	28	Sativex	om	12 sprays per day	32.4 mg	M.D.	9h
S02-02	m	54	28	Sativex	om	6 to 8 sprays per day	16.2-21.6 mg	2 sprays (5.4 mg THC)	16h 15min
S03-01	f	55	28	Sativex	om	4 - 0 - 6 sprays	27 mg	4 sprays (10.8 mg THC)	50 min
S03-02	f	56	29	Sativex	om	4 - 0 - 12 sprays	43.2 mg	4 sprays (10.8 mg THC)	33 min
S03-03	f	56	29	Sativex	om	4 - 0 - 6 sprays	27 mg	4 sprays (10.8 mg THC)	1h 24min
S03-04	f	56	29	Sativex	om	8 - 0 - 12 sprays	54 mg	4 sprays (10.8 mg THC)	1h 5min
S04-01	f	51	24	Sativex + Bedrocan	om, M.D.	0 - 0 - 2 to 3 sprays + unknown quantity of Bedrocan	5.4 to 8.1 mg + unknown quantity of THC from Bedrocan	2 to 3 sprays (5.4 to 8.1 mg THC)	10h 44min (Sativex)
S05-01	m	55	37	Sativex + Bedrocan	om, v*	Sativex: 1 - 0 - 2 sprays, Bedrocan: 500 mg at noon	118.1 mg (= 8.1 mg (Sativex) + 110 mg (Bedrocan))	2 sprays (5.4 mg THC)	13h 35min (Sativex)
S05-02	m	55	36	Sativex + Bedrocan	om, v	Sativex: 1 - 0 - 2 sprays, Bedrocan: 100 mg at noon	30.1 mg (= 8.1 mg (Sativex) + 22 mg (Bedrocan))	1 spray (2.7 mg THC)	2h 15min (Sativex)
S05-03	m	55	35	Sativex + Bedrocan	om, v	Sativex: 1 - 0 - 2 sprays, Bedrocan: 100 mg at noon	30.1 mg (= 8.1 mg (Sativex) + 22 mg (Bedrocan))	1 spray (2.7 mg THC)	1h 45min (Sativex)
S05-04	m	55	35	Sativex + Bedrocan	om, v	Sativex: 1 - 0 - 3 sprays, Bedrocan: 100 mg at noon	32.8 mg (= 10.8 mg (Sativex) + 22 mg (Bedrocan))	1 spray (2.7 mg THC)	3h 30min (Sativex)
S05-05	m	55	34	Sativex + Bedrocan	om, v	Sativex: 1 - 0 - 3 sprays, Bedrocan: 100 mg at noon	32.8 mg (= 10.8 mg (Sativex) + 22 mg (Bedrocan))	1 spray (2.7 mg THC)	2h 10min (Sativex)
S06-01	m	27	22	Bediol	v	50 mg - 50 mg - 50 mg	9.45 mg	50 mg (3.15mg THC)	3h 56min

Table S1. Cont.

Sample	Gender	Age	BMI*	Substance intake	Application	Dosing regimen per day	Approx. daily dose of THC [mg]***	Last dose before blood draw (respective THC quantity)	Time between last intake and blood draw
S07-01	m	58	25	Bedrocan	s*, v	3 g (10 doses at 300 mg each)	660 mg	300 mg (66 mg THC)	1h 10min
S07-02	m	58	24	Bedrocan	s, v	2.5 to 3 g (10 doses at 250 to 300 mg each, every 2 to 3 hours)	550 to 660 mg	250 to 300 mg (55 to 66 mg THC)	1h 30min
S07-03	m	58	23	Bedrocan	v	2 to 2.5 g (10 doses at 200 to 250 mg each, every 2 hours)	440 to 550 mg	200 to 250 mg (44 to 55 mg THC)	2h 30min
S07-04	m	59	23	Bedrocan	v	2.5 g (10 doses at 250mg each)	550 mg	250 mg (55 mg THC)	1h 10min
S08-01	f	36	54	Bedrolite + Pedanios 22/1	v	100 mg Bedrolite - 100 mg Bedrolite - 100 mg Pedanios 22/1	23.98 mg (= 1.98 mg (Bedrolite) + 22 mg (Pedanios 22/1))	100mg Pedanios 22/1 (22mg THC)	10h 10min (Pedanios 22/1)
S08-02	f	36	55	Bedrolite + Bedrocan	v	100 mg Bedrolite - 100 mg Bedrolite - 100 mg Bedrocan	23.98 mg (= 1.98 mg (Bedrolite) + 22 mg (Bedrocan))	100mg Bedrolite (0.99 mg THC)	9h (Bedrolite)
S08-03	f	36	54	Bedrolite + Bedrocan	v	70 mg Bedrolite - 70 mg Bedrolite - 70 mg Bedrocan	16.79 mg (= 1.39 mg (Bedrolite) + 15.4 mg (Bedrocan))	70mg Bedrolite (0.69 mg THC)	2h 30min (Bedrolite)
S08-04	f	36	54	Bedrolite + Bedrocan	v	100 mg Bedrocan - 0 - 100 mg Bedrolite (+ 100 mg Bedrocan before bedtime if necessary)	44.99 mg (= 0.99 mg (Bedrolite) + 44 mg (Bedrocan))	100mg Bedrocan (22 mg THC)	2h 30min (Bedrocan)
S09-01	m	56	31	Bedrocan	v	0 - 0 - 50 mg	11 mg	50 mg (11 mg THC)	15h 26min
S09-02	m	57	M.D.	Bedrocan	v	0 - 0 - 50 mg + 50 mg before bedtime	22 mg	50 mg (11 mg THC)	10h 55min
S09-03	m	57	28	Bedrocan	v	50 mg every second day/ if necessary	11 mg	50 mg (11 mg THC)	1d 14h 50min
S10-01	m	49	29	Bedrobinol	s	1 g (divided into 4 to 6 doses)	135 mg	166-250 mg (22.41-33.75 mg THC)	4h 50min
S10-02	m	50	30	Bedrobinol	s	1 g (divided into 4 to 6 doses)	135 mg	166-250 mg (22.41-33.75 mg THC)	3h 20min
S10-03	m	50	30	Bedrobinol	s	6 doses of 200 mg each	162 mg	200 mg (27 mg THC)	1h 55min
S10-04	m	50	30	Bedrobinol	s	4 to 6 doses of 200 mg each	108 to 162 mg	200 mg (27 mg THC)	5d 14h 40min

Table S1. Cont.

Sample	Gender	Age	BMI*	Substance intake	Application	Dosing regimen per day	Approx. daily dose of THC [mg]***	Last dose before blood draw (respective THC quantity)	Time between last intake and blood draw
S11-01	m	23	23	Dronabinol + Street cannabis	o*, s	6 - 6 - 6 drops of Dronabinol + 3-4 joints	15.84 mg (Dronabinol) + unknown quantity of THC (joint)	6 drops (5.28 mg THC)	6h 34min (Dronabinol)
S11-02	m	23	23	Dronabinol + Street cannabis	o, s	6 - 6 - 6 drops of Dronabinol + unknown number of joints	15.84 mg (Dronabinol) + unknown quantity of THC (joint)	6 drops (5.28 mg THC)	8h 19min (Dronabinol)
S11-03	m	23	22	Dronabinol + Street cannabis	o, s	unknown number of joints	M.D.	M.D.	approx. 4.5d (Dronabinol)
S11-04	m	23	21	Dronabinol + Street cannabis	o, s	1 joint - 1 joint - 1 joint, Dronabinol intake discontinued for 11 days	M.D.	1 joint (M.D.)	12h 50min (joint)
S12-01	m	58	24	Dronabinol + treet cannabis	o, s	10 - 10 - 10 drops of Dronabinol + 3 joints	26.4 mg (Dronabinol) + unknown quantity of THC (joint)	10 drops (8.8 mg THC)	3h (Dronabinol)
S12-02	m	58	25	Dronabinol + Street cannabis	o, v	12 - 12 - 15 drops of Dronabinol + unknown quantity of cannabis	34.32 mg (Dronabinol) + unknown quantity of THC (cannabis)	12 drops (10.56 mg THC)	2h 47min (Dronabinol)
S13-01	f	75	29	Dronabinol	o	3 - 0 - 3 drops	5.28 mg	5 drops (4.4 mg THC)	7h 20min
S14-01	f	78	17	Dronabinol	o	2 doses of 2 to 3 drops each	3.52-5.28 mg	2-3 drops (1.76-2.64 mg THC)	1h 20min
S15-01	m	76	23	Dronabinol	o	5 - 0 - 5 drops	8.80 mg	5 drops (4.4 mg THC)	1h 40min
S15-02	m	76	22	Dronabinol	o	2 doses to 6 to 8 drops each	10.56-14.08 mg	6-8 drops (5.28-7.04 mg THC)	3h 20min
S16-01	m	59	31	Dronabinol	o	4 - 0 - 0 drops	3.52 mg	4 drops (3.52 mg THC)	23h 40min
S16-02	m	60	31	Dronabinol	o	6 - 0 - 0 drops	5.28 mg	6 drops (5.28 mg THC)	24h 30min
S16-03	m	60	31	Dronabinol	o	6 - 0 - 0 drops	5.28 mg	6 drops (5.28 mg THC)	3h 17min

Table S1. Cont.

Sample	Gender	Age	BMI*	Substance intake	Application	Dosing regimen per day	Approx. daily dose of THC [mg]***	Last dose before blood draw (respective THC quantity)	Time between last intake and blood draw
S17-01	f	54	24	Dronabinol	o	5 - 0 - 10 drops	13.2 mg	5 drops (4.4 mg THC)	6h 20min
S17-02	f	55	24	Dronabinol	o	3 -0 - 10 drops	11.44 mg	3 drops (2.64 mg THC)	6h 45min
S17-03	f	55	24	Dronabinol	o	0 - 0 - 5 drops	4.4 mg	5 drops (4.4 mg THC)	15h 6min
S17-04	f	55	24	Dronabinol	o	0 - 0 - 5 drops	4.4 mg	5 drops (4.4 mg THC)	15h 30min
S17-05	f	55	24	Dronabinol	o	0 - 0 - 5 drops	4.4 mg	5 drops (4.4 mg THC)	15h 25min
S18-01	f	64	15	Dronabinol	o	5 - 5 - 5 drops	13.2 mg	5 drops (4.4 mg THC)	2h 5min
S19-01	f	64	20	Dronabinol	o	6 - 6 - 6 drops	15.84 mg	6 drops (5.28 mg THC)	1h
S19-02	f	64	20	Dronabinol	o	'intake discontinued for some days'	M.D.	M.D.	M.D.
S20-01	f	46	24	Dronabinol	o	2 - 0 - 2 drops	3.52 mg	2 drops (1.76 mg THC)	1 to 1,5h
S21-01	f	56	25	Dronabinol	o	5 - 5 - 5 drops	13.2 mg	5 drops (4.4 mg THC)	3h 24min
S22-01	f	55	33	Dronabinol	o	0 - 0 - 4 drops	3.52 mg	4 drops (3.52 mg THC)	13h 54min
S23-01	f	44	23	Dr. Nice CBD capsules	o	0 - 0 - 1 capsule	1.64 mg	1 capsule (1.64 mg)	14h 15min

* f = female, m = male, BMI = body mass index, om = oromucosal, v = vaporized, s = smoked, o = oral, M.D. = missing data

** 3 doses were applied in the morning, 0 doses at noon/afternoon and 3 doses in the evening

*** Calculated as follows: 1 spray Sativex ≙ 2.7 mg THC [7], Pedanios 22/1 ≙ 22% THC [41], Bedrocan ≙ 22% THC, Bediol ≙ 6.3% THC, Bedrolite ≙ 0.99% (<1.0%) THC, Bedrobinol ≙ 13.5% THC [42], 1 drop of Dronabinol ≙ 0.88 mg THC [43], 1 Dr. Nice CBD capsule ≙ 1.64 mg THC (Extract in the capsules contains 34.4 mg CBD/g and 1.88 mg THC/g [17]. 1 capsule contains at least 30 mg CBD, which equals approx. 30 mg CBD / 34.4 mg CBD/g extract = 0.87g. 0.87g extract ≙ 0.87 g * 1.88 mg THC/g = 1.64 mg THC).

Table S2. Cannabinoid concentrations of forensic serum samples analyzed in a previous study [16] in [ng/mL]

Sample	Substance intake	THC	THC-OH	THC-COOH	THCAA	CBD	CBDA	CBN	CBNA	CBG	CBGA	CBC	CBCA	CBL	CBLA	THCV	THCVA	CBDV	CBDVA
F01	Street cannabis	10	2.9	27	10	nd	(0.0028)*	0.22	0.018	0.44	0.51	1.3	0.75	nd	0.010	(0.032)	1.7	nd	nd
F02	Street cannabis	42	14	180	9.8	0.55	nd	1.9	0.089	0.85	0.13	4.4	0.20	nd	nd	0.24	1.0	nd	nd
F03	Street cannabis	0.36	0.44	8.3	0.24	0.40	0.12	0.015	0.034	nd	0.037	0.20	0.035	nd	nd	nd	0.16	nd	(0.0063)
F04	Street cannabis	1.3	1.0	18	0.27	(0.054)	nd	0.042	(0.0044)	(0.055)	0.022	0.20	nd	nd	nd	nd	0.28	nd	nd
F05	Street cannabis	4.0	1.5	22	0.26	0.57	0.011	0.52	(0.0092)	0.17	0.031	0.52	(0.018)	nd	nd	(0.048)	0.45	nd	nd
F06	Street cannabis	1.2	0.76	8.6	0.078	(0.042)	nd	0.044	(0.0061)	(0.040)	(0.013)	0.26	(0.0080)	nd	nd	nd	0.18	nd	nd
F07	Street cannabis	0.20	nd	10	0.020	nd	nd	(0.0055)	nd	nd	nd	nd	nd	nd	nd	nd	0.040	nd	nd
F08	Street cannabis	2.0	1.0	14	0.28	(0.050)	nd	0.094	(0.0057)	(0.062)	(0.0084)	0.20	(0.014)	nd	nd	nd	0.075	nd	nd
F09	Street cannabis	0.82	0.78	15	0.056	nd	nd	0.024	(0.0038)	nd	nd	(0.10)	nd	nd	nd	nd	0.070	nd	nd
F10	Street cannabis	1.2	(0.26)	4.1	0.12	nd	nd	0.031	(0.0082)	(0.048)	nd	nd	nd	nd	nd	nd	0.052	nd	nd
F11	Street cannabis	3.5	1.1	25	0.66	nd	nd	0.11	(0.015)	0.15	0.024	0.34	(0.015)	nd	nd	nd	0.27	nd	nd
F12	Street cannabis	1.1	0.81	47	0.15	nd	nd	0.023	(0.0064)	(0.058)	0.023	(0.10)	(0.0099)	nd	nd	nd	0.077	nd	nd
F13	Street cannabis	0.68	0.40	7.4	0.043	0.17	nd	0.041	(0.0069)	nd	(0.0093)	(0.10)	(0.0094)	nd	nd	nd	0.041	nd	nd
F14	Street cannabis	0.83	0.77	19	0.068	nd	nd	0.012	(0.011)	nd	(0.0093)	(0.10)	nd	nd	nd	nd	0.063	nd	nd
F15	Street cannabis	0.60	0.40	2.0	0.028	nd	nd	0.031	(0.0059)	nd	nd	(0.10)	nd	nd	nd	nd	(0.027)	nd	nd
F16	Street cannabis	0.45	nd	3.0	0.047	nd	nd	0.013	(0.0060)	nd	nd	nd	nd	nd	nd	nd	0.047	nd	nd
F17	Street cannabis	13	17	180	9.9	0.42	0.024	0.30	0.023	0.26	0.41	1.7	0.15	nd	0.010	(0.020)	0.78	nd	nd
F18	Street cannabis	55	9.3	160	64	0.14	0.0059	1.6	0.18	1.4	1.7	5.6	3.1	0.19	0.011	0.71	51	nd	nd
F19	Street cannabis	31	21	170	11	0.13	nd	0.51	0.027	1.8	0.74	2.3	0.25	nd	0.010	0.20	2.3	nd	nd
F20	Street cannabis	26	12	190	19	nd	nd	0.51	0.056	1.7	2.0	2.3	0.59	nd	0.013	(0.15)	1.4	nd	nd
F21	Street cannabis	(0.055)	nd	2.9	0.23	nd	nd	0.010	nd	nd	(0.013)	nd	(0.026)	nd	(0.0022)	nd	1.5	nd	nd
F22	Street cannabis	3.3	2.0	26	0.47	nd	nd	0.077	(0.015)	0.25	0.083	0.60	0.054	nd	nd	nd	0.21	nd	nd
F23	Street cannabis	0.31	(0.20)	(1.2)	0.070	nd	nd	0.0085	(0.0056)	nd	(0.011)	nd	(0.012)	nd	nd	nd	(0.033)	nd	nd
F24	Street cannabis	0.75	(0.35)	11	0.065	nd	nd	0.042	(0.0073)	nd	(0.0092)	(0.10)	nd	nd	nd	nd	0.063	nd	nd

Table S2. Cont.

Sample	Substance intake	THC	THC-OH	THC-COOH	THCAA	CBD	CBDA	CBN	CBNA	CBG	CBGA	CBC	CBCA	CBL	CBLA	THCV	THCVA	CBDV	CBDVA
F25	Street cannabis	0.24	nd	3.0	0.033	nd	nd	0.0094	0.016	nd	nd	nd	nd	nd	nd	nd	0.042	nd	nd
F26	Street cannabis	3.7	2.7	93	0.27	nd	nd	0.18	0.025	(0.075)	0.034	0.52	(0.025)	(0.071)	(0.0020)	nd	2.2	nd	nd
F27	Street cannabis	3.2	2.8	80	7.0	1.5	0.15	0.097	0.020	(0.066)	0.28	0.51	0.17	nd	(0.0048)	nd	0.79	nd	nd
F28	Street cannabis	0.81	0.46	19	0.64	nd	(0.0022)	0.021	0.028	nd	0.021	nd	(0.021)	nd	nd	nd	0.073	nd	nd
F29	Street cannabis	1.3	1.8	38	0.048	(0.069)	nd	0.062	(0.0051)	nd	(0.014)	(0.10)	nd	nd	nd	nd	(0.033)	nd	nd
F30	Street cannabis	1.7	1.3	51	0.32	nd	nd	0.030	(0.0069)	0.083	(0.011)	0.20	(0.017)	nd	nd	nd	0.088	nd	nd
F31	Street cannabis	0.63	(0.33)	21	(0.0099)	nd	nd	0.0080	(0.0061)	nd	nd	nd	nd	nd	nd	nd	0.049	nd	nd
F32	Street cannabis	2.5	1.9	28	2.7	nd	nd	0.042	(0.0083)	0.10	0.10	0.22	(0.026)	nd	nd	nd	0.95	nd	nd
F33	Street cannabis	0.41	nd	3.0	0.18	nd	nd	(0.0053)	(0.0040)	nd	nd	nd	nd	nd	nd	nd	0.13	nd	nd
F34	Street cannabis	0.47	(0.27)	3.7	0.026	nd	nd	0.0091	(0.0044)	nd	nd	nd	nd	nd	nd	nd	0.81	nd	nd
F35	Street cannabis	2.5	0.78	40	0.090	nd	nd	0.056	nd	(0.040)	nd	(0.11)	nd	nd	nd	nd	0.52	nd	nd
F36	Street cannabis	0.94	0.54	20	0.035	nd	nd	0.026	nd	nd	nd	(0.10)	nd	nd	nd	nd	0.64	nd	nd
F37	Street cannabis	0.34	nd	2.0	(0.0095)	nd	nd	(0.0067)	(0.0041)	nd	nd	nd	nd	nd	nd	nd	0.12	nd	nd
F38	Street cannabis	1.6	0.93	32	0.092	nd	nd	0.038	(0.0052)	0.076	nd	(0.10)	nd	nd	nd	(0.021)	0.42	nd	nd
F39	Street cannabis	0.80	0.40	7.7	0.029	nd	nd	0.022	(0.0050)	nd	nd	(0.10)	nd	nd	nd	(0.020)	0.27	nd	nd
F40	Street cannabis	0.28	nd	3.0	(0.0059)	nd	nd	(0.0041)	nd	nd	nd	nd	nd	nd	nd	nd	0.16	nd	nd
F41	Street cannabis	0.61	(0.27)	4.4	(0.012)	nd	nd	0.011	(0.0060)	(0.040)	nd	nd	nd	nd	nd	nd	0.11	nd	nd
F42	Street cannabis	0.44	0.40	(1.0)	0.029	nd	nd	(0.0027)	nd	nd	nd	nd	nd	nd	nd	nd	0.067	nd	nd
F43	Street cannabis	8.6	5.3	200	0.031	0.47	(0.0029)	0.19	(0.0052)	0.31	(0.014)	0.20	(0.0080)	nd	nd	(0.10)	0.65	nd	nd
F44	Street cannabis	0.47	(0.20)	4.6	(0.0013)	nd	nd	0.018	(0.0047)	(0.040)	nd	nd	nd	nd	nd	nd	0.16	nd	nd
F45	Street cannabis	0.60	0.40	11	0.16	nd	nd	0.011	nd	nd	nd	nd	nd	nd	nd	nd	0.19	nd	nd
F46	Street cannabis	0.64	0.41	3.0	0.22	nd	nd	0.012	nd	nd	(0.0093)	nd	(0.0090)	nd	nd	nd	0.53	nd	nd
F47	Street cannabis	1.0	(0.30)	3.9	0.021	nd	nd	0.024	(0.0043)	(0.058)	nd	nd	nd	nd	nd	nd	0.046	nd	nd
F48	Street cannabis	0.46	(0.25)	3.7	0.031	nd	nd	0.0092	nd	nd	nd	nd	nd	nd	nd	nd	0.11	nd	nd

Table S2. Cont.

Sample	Substance intake	THC	THC-OH	THC-COOH	THCAA	CBD	CBDA	CBN	CBNA	CBG	CBGA	CBC	CBCA	CBL	CBLA	THCV	THCVA	CBDV	CBDVA
F49	Street cannabis	0.89	0.40	7.1	0.027	nd	nd	0.012	nd	nd	nd	nd	nd	nd	nd	nd	0.35	nd	nd
F50	Street cannabis	0.20	nd	(1.0)	0.44	nd	nd	(0.0018)	0.017	nd	nd	nd	(0.012)	nd	nd	nd	0.20	nd	nd
F51	Street cannabis	0.45	nd	5.4	0.028	nd	nd	0.010	(0.0041)	nd	nd	nd	nd	nd	nd	nd	0.13	nd	nd
F52	Street cannabis	0.29	(0.20)	2.0	(0.010)	nd	nd	(0.0060)	nd	nd	nd	nd	nd	nd	nd	nd	0.049	nd	nd
F53	Street cannabis	0.37	(0.20)	5.5	0.020	nd	nd	0.022	nd	nd	nd	nd	nd	nd	nd	nd	0.076	nd	nd
F54	Street cannabis	0.20	nd	(1.0)	0.13	nd	nd	(0.0013)	nd	nd	(0.0096)	nd	nd	nd	nd	nd	0.045	nd	nd
F55	Street cannabis	0.56	(0.30)	7.5	0.13	nd	nd	0.010	nd	nd	0.020	nd	nd	nd	nd	nd	0.25	nd	nd

* Measured values <LOQ are reported as approximate values in parentheses, nd = not detected/ below limit of detection

Table S3. Cannabinoid serum concentrations of study and forensic serum samples standardized on THC (Cannabinoid ratios)

Sample	Substance intake	$\frac{THC-OH}{THC}$	$\frac{THC-COOH}{THC}$	$\frac{THCAA}{THC}$	$\frac{CBD}{THC}$	$\frac{CBDA}{THC}$	$\frac{CBN}{THC}$	$\frac{CBNA}{THC}$	$\frac{CBG}{THC}$	$\frac{CBGA}{THC}$	$\frac{CBC}{THC}$	$\frac{CBCA}{THC}$	$\frac{CBL}{THC}$	$\frac{CBLA}{THC}$	$\frac{THCV}{THC}$	$\frac{THCVA}{THC}$	$\frac{CBDVA}{THC}$
S01-01	Sativex	2.62	76.5	0.0471	1.06	0	0.0250	0	0	0	0.412	0	0	0	0	0.0677	0
S01-02	Sativex	2.82	86.4	0.0546	1.14	0	0.0146	0	0	0	0	0	0	0	0	0.0864	0
S01-03	Sativex	2.40	127	0.0367	1.53	0	0.0107	0	0	0	0	0	0	0	0	0.140	0
S02-01	Sativex	1.09	20.9	0.0255	0.682	0.00182	0.0182	0	0	0	0.100	0	0	0	0	0.0209	0
S02-02	Sativex	3.46	41.5	0.0362	0.770	0.00215	0.0231	0	0	0	0.223	0	0	0	0	0.0162	0
S03-01	Sativex	8.90	58.5	0.0683	1.22	0.0122	0.0293	0	0	0	0.342	0	0	0	0	0.0183	0
S03-02	Sativex	10.2	114	0.0508	1.22	0.00857	0.0254	0	0	0	0.254	0	0	0	0	0.0270	0
S03-03	Sativex	2.61	21.7	0.0183	0.609	0.00891	0.00957	0	0	0	0.178	0.00652	0	0	0	0.00413	0
S03-04	Sativex	5.00	38.6	0.00773	1.39	0.00955	0.00909	0.00150	0	0.00250	0.250	0.00727	0	0	0	0.00546	0
S04-01	Sativex+Bedrocan	0.450	1.50	0	0.371	0.00929	0.0121	0	0	0	0.0714	0.00786	0	0	0	0.0171	0
S05-01	Sativex+Bedrocan	0	68.0	0.0620	0	0	0.0220	0	0	0	0	0	0	0	0	0.340	0
S05-02	Sativex+Bedrocan	2.33	51.2	0.0395	0.605	0.00977	0.0181	0	0	0	0.233	0	0	0	0	0.0744	0
S05-03	Sativex+Bedrocan	1.96	66.7	0.0889	0.756	0.0533	0.0267	0	0	0	0.267	0	0	0	0	0.104	0
S05-04	Sativex+Bedrocan	2.18	50.9	0.0327	0.582	0.00646	0.0182	0	0	0	0.255	0.0136	0	0	0	1.64	0
S05-05	Sativex+Bedrocan	1.97	38.0	0.0563	0.747	0.00479	0.0141	0	0	0	0.254	0	0	0	0	0.0409	0
S06-01	Bediol	0.766	7.66	0.0138	2.13	0.00575	0.0511	0	0	0	0.479	0	0	0	0	0.192	0
S07-01	Bedrocan	0.449	3.47	0.190	0.00367	0.000180	0.0198	0.000449	0.0490	0.0225	0.0918	0.0118	0	0.000449	0.00735	0.0653	0
S07-02	Bedrocan	0.531	5.31	0.116	0.00222	0.0000969	0.0150	0.000406	0.0469	0.0169	0.0938	0.00656	0	0.000300	0.00719	0.0844	0
S07-03	Bedrocan	0.605	3.58	0.0442	0.00186	0.0000814	0.0214	0.000698	0.0395	0.0172	0.0977	0.00837	0	0.000326	0.00581	0.0212	0
S07-04	Bedrocan	0.438	3.75	0.0333	0.000938	0.000110	0.0175	0.000542	0.0438	0.00708	0.0917	0.00250	0	0.000148	0.00354	0.0313	0
S08-01	Bedrolite+Pedanios	-	-	-	-	-	-	-	-	-	-	-	-	-	-	-	-
S08-02	Bedrolite+Bedrocan	-	-	-	-	-	-	-	-	-	-	-	-	-	-	-	-
S08-03	Bedrolite+Bedrocan	-	-	-	-	-	-	-	-	-	-	-	-	-	-	-	-
S08-04	Bedrolite+Bedrocan	0	0	0.0613	0	0	0	0	0	0	0	0	0	0	0	0	0

Table S3. Cont.

Sample	Substance intake	$\frac{THC-OH}{THC}$	$\frac{THC-COOH}{THC}$	$\frac{THCAA}{THC}$	$\frac{CBD}{THC}$	$\frac{CBDA}{THC}$	$\frac{CBN}{THC}$	$\frac{CBNA}{THC}$	$\frac{CBG}{THC}$	$\frac{CBGA}{THC}$	$\frac{CBC}{THC}$	$\frac{CBCA}{THC}$	$\frac{CBL}{THC}$	$\frac{CBLA}{THC}$	$\frac{THCV}{THC}$	$\frac{THCVA}{THC}$	$\frac{CBDVA}{THC}$
S09-01	Bedrocan	1.08	28.2	0.0239	0.0923	0.0162	0.0462	0	0	0.0564	0	0	0	0	0	0.949	0
S09-02	Bedrocan	0.643	15.2	0.112	0	0.00476	0.0286	0	0	0.0571	0	0	0	0	0	0.548	0
S09-03	Bedrocan	0	12.9	0.112	0	0	0.112	0	0	0	0	0	0	0	0	1.59	0
S10-01	Bedrobinol	0	0	0.0600	0	0	0.0180	0	0	0	0	0	0	0	0	0.0435	0
S10-02	Bedrobinol	0.405	3.78	0.238	0.0235	0	0.0676	0	0.0243	0.00892	0.173	0.00784	0	0	0	0.0566	0
S10-03	Bedrobinol	0.426	4.26	0.277	0.0162	0	0.0383	0	0.0168	0.0115	0.181	0.0117	0	0	0	0.119	0
S10-04	Bedrobinol	0.524	4.00	0.214	0.0348	0	0.0571	0	0.0219	0.00524	0.129	0.0110	0	0	0	0.133	0
S11-01	Dronabinol + Street cannabis	1.19	25.0	0.0191	0	0	0.0321	0	0	0.00976	0.179	0	0	0.00250	0	0.345	0
S11-02	Dronabinol + Street cannabis	0.714	15.0	0.121	0	0	0.0207	0.00350	0.0407	0.0171	0.129	0.00929	0	0	0	1.64	0
S11-03	Dronabinol + Street cannabis	0.634	6.10	0.268	0	0	0.0342	0.0110	0	0.0293	0	0.0268	0	0.00585	0	0.756	0
S11-04	Dronabinol + Street cannabis	0.575	15.8	0.0917	0	0	0.0267	0	0.0467	0.0125	0.225	0.00833	0	0.00192	0	0.333	0
S12-01	Dronabinol+Street cannabis	0.482	26.4	0.0600	0	0	0.0318	0	0.0755	0.0127	0.118	0.00227	0	0	0	0.0709	0
S12-02	Dronabinol+Street cannabis	0.750	13.1	0.0488	0.00275	0	0.0294	0.00125	0.0413	0.00688	0.0750	0.00200	0	0	0	0.106	0
S13-01	Dronabinol	3.96	47.9	0.00104	0	0	0	0	0	0.0190	0	0	0	0	0	0	0
S14-01	Dronabinol	1.13	19.5	0.000488	0	0	0.00927	0	0	0	0	0	0	0	0	0.220	0
S15-01	Dronabinol	3.12	76.5	0	0	0	0	0	0	0	0	0	0	0	0	0	0
S15-02	Dronabinol	2	29.0	0	0	0	0.00240	0	0	0.00850	0	0	0	0	0	0	0

Table S3. Cont.

Sample	Substance intake	$\frac{THC-OH}{THC}$	$\frac{THC-COOH}{THC}$	$\frac{THCAA}{THC}$	$\frac{CBD}{THC}$	$\frac{CBDA}{THC}$	$\frac{CBN}{THC}$	$\frac{CBNA}{THC}$	$\frac{CBG}{THC}$	$\frac{CBGA}{THC}$	$\frac{CBC}{THC}$	$\frac{CBCA}{THC}$	$\frac{CBL}{THC}$	$\frac{CBLA}{THC}$	$\frac{THCV}{THC}$	$\frac{THCVA}{THC}$	$\frac{CBDVA}{THC}$
S16-01	Dronabinol	1.33	16.0	0.0167	0	0	0	0	0	0	0	0	0	0	0	0	0
S16-02	Dronabinol	0	48.0	0	0	0	0	0	0	0	0	0	0	0	0	0	0
S16-03	Dronabinol	2.83	28.7	0.0739	0	0	0	0	0	0	0	0	0	0	0	0	0
S17-01	Dronabinol	1.08	25.2	0.0600	0	0	0	0	0	0	0	0	0	0	0	0	0
S17-02	Dronabinol	10.1	186	0.243	0	0	0.0314	0	0	0	0	0	0	0	0	0	0
S17-03	Dronabinol	-	-	-	-	-	-	-	-	-	-	-	-	-	-	-	-
S17-04	Dronabinol	-	-	-	-	-	-	-	-	-	-	-	-	-	-	-	-
S17-05	Dronabinol	-	-	-	-	-	-	-	-	-	-	-	-	-	-	-	-
S18-01	Dronabinol	0	0	0.0367	0	0	0	0	0	0	0	0	0	0	0	0	0
S19-01	Dronabinol	5.56	15.6	0.172	0	0	0.0294	0	0	0	0	0	0	0	0	0	0
S19-02	Dronabinol	-	-	-	-	-	-	-	-	-	-	-	-	-	-	-	-
S20-01	Dronabinol	0	20.0	0	0	0	0.0124	0	0	0	0	0	0	0	0	0	0
S21-01	Dronabinol	3.91	40.6	0.0116	0	0	0.00667	0.00783	0	0	0	0	0	0	0	0	0
S22-01	Dronabinol	3.07	107	0	0	0	0	0	0	0	0	0	0	0	0	0.213	0
S23-01	CBD capsules	-	-	-	-	-	-	-	-	-	-	-	-	-	-	-	-
F01	Street cannabis	0.290	2.70	1.00	0	0.000280	0.0220	0.00180	0.0440	0.0510	0.130	0.0750	0	0.00100	0.00320	0.170	0
F02	Street cannabis	0.333	4.29	0.233	0.0131	0	0.0452	0.00212	0.0202	0.00310	0.105	0.00476	0	0	0.00571	0.0238	0
F03	Street cannabis	1.22	23.1	0.667	1.11	0.333	0.0417	0.0944	0	0.103	0.556	0.0972	0	0	0	0.444	0.0175
F04	Street cannabis	0.769	13.9	0.208	0.0415	0	0.0323	0.00339	0.0423	0.0169	0.154	0	0	0	0	0.215	0
F05	Street cannabis	0.375	5.50	0.0650	0.143	0.00275	0.130	0.00230	0.0425	0.00775	0.130	0.00450	0	0	0.0120	0.113	0
F06	Street cannabis	0.633	7.17	0.0650	0.0350	0	0.0367	0.00509	0.0333	0.0108	0.217	0.00667	0	0	0	0.150	0
F07	Street cannabis	0	50.0	0.100	0	0	0.0275	0	0	0	0	0	0	0	0	0.200	0

Table S3. Cont.

Sample	Substance intake	$\frac{THC-OH}{THC}$	$\frac{THC-COOH}{THC}$	$\frac{THCAA}{THC}$	$\frac{CBD}{THC}$	$\frac{CBDA}{THC}$	$\frac{CBN}{THC}$	$\frac{CBNA}{THC}$	$\frac{CBG}{THC}$	$\frac{CBGA}{THC}$	$\frac{CBC}{THC}$	$\frac{CBCA}{THC}$	$\frac{CBL}{THC}$	$\frac{CBLA}{THC}$	$\frac{THCV}{THC}$	$\frac{THCVA}{THC}$	$\frac{CBDVA}{THC}$
F08	Street cannabis	0.500	7.00	0.140	0.0250	0	0.0470	0.00285	0.0310	0.00420	0.100	0.00700	0	0	0	0.0375	0
F09	Street cannabis	0.951	18.3	0.0683	0	0	0.0293	0.00463	0	0	0.122	0	0	0	0	0.0854	0
F10	Street cannabis	0.217	3.42	0.100	0	0	0.0258	0.00683	0.0400	0	0	0	0	0	0	0.0433	0
F11	Street cannabis	0.314	7.14	0.189	0	0	0.0314	0.00429	0.0429	0.00686	0.0971	0.00429	0	0	0	0.0771	0
F12	Street cannabis	0.736	42.7	0.136	0	0	0.0209	0.00582	0.0527	0.0209	0.0909	0.00900	0	0	0	0.0700	0
F13	Street cannabis	0.588	10.9	0.0632	0.250	0	0.0603	0.0102	0	0.0137	0.147	0.0138	0	0	0	0.0603	0
F14	Street cannabis	0.928	22.9	0.0819	0	0	0.0145	0.0133	0	0.0112	0.121	0	0	0	0	0.0759	0
F15	Street cannabis	0.667	3.33	0.0467	0	0	0.0517	0.00983	0	0	0.167	0	0	0	0	0.0450	0
F16	Street cannabis	0	6.67	0.104	0	0	0.0289	0.0133	0	0	0	0	0	0	0	0.104	0
F17	Street cannabis	1.31	13.9	0.762	0.0323	0.00185	0.0231	0.00177	0.0200	0.0315	0.131	0.0115	0	0.000769	0.00154	0.0600	0
F18	Street cannabis	0.169	2.91	1.16	0.00255	0.000107	0.0291	0.00327	0.0255	0.0309	0.102	0.0564	0.00346	0.000200	0.0129	0.927	0
F19	Street cannabis	0.677	5.48	0.355	0.00419	0	0.0165	0.000871	0.0581	0.0239	0.0742	0.00807	0	0.000323	0.00645	0.0742	0
F20	Street cannabis	0.462	7.31	0.731	0	0	0.0196	0.00215	0.0654	0.0769	0.0885	0.0227	0	0.000500	0.00577	0.0539	0
F21	Street cannabis	0	52.7	4.18	0	0	0.182	0	0	0.236	0	0.473	0	0.0400	0	27.3	0
F22	Street cannabis	0.606	7.88	0.142	0	0	0.0233	0.00455	0.0758	0.0252	0.182	0.0164	0	0	0	0.0636	0
F23	Street cannabis	0.645	3.87	0.226	0	0	0.0274	0.0181	0	0.0355	0	0.0387	0	0	0	0.107	0
F24	Street cannabis	0.467	14.7	0.0867	0	0	0.0560	0.00973	0	0.0123	0.133	0	0	0	0	0.0840	0
F25	Street cannabis	0	12.5	0.138	0	0	0.0392	0.0667	0	0	0	0	0	0	0	0.175	0
F26	Street cannabis	0.730	25.1	0.0730	0	0	0.0487	0.00676	0.0203	0.00919	0.141	0.00676	0.0192	0.000541	0	0.595	0
F27	Street cannabis	0.875	25.0	2.19	0.469	0.0469	0.0303	0.00625	0.0206	0.0875	0.159	0.0531	0	0.00150	0	0.247	0
F28	Street cannabis	0.568	23.5	0.790	0	0.00272	0.0259	0.0346	0	0.0259	0	0.0259	0	0	0	0.0901	0
F29	Street cannabis	1.39	29.2	0.0369	0.0531	0	0.0477	0.00392	0	0.0108	0.0769	0	0	0	0	0.0254	0

Table S3. Cont.

Sample	Substance intake	$\frac{THC-OH}{THC}$	$\frac{THC-COOH}{THC}$	$\frac{THCAA}{THC}$	$\frac{CBD}{THC}$	$\frac{CBDA}{THC}$	$\frac{CBN}{THC}$	$\frac{CBNA}{THC}$	$\frac{CBG}{THC}$	$\frac{CBGA}{THC}$	$\frac{CBC}{THC}$	$\frac{CBCA}{THC}$	$\frac{CBL}{THC}$	$\frac{CBLA}{THC}$	$\frac{THCV}{THC}$	$\frac{THCVA}{THC}$	$\frac{CBDVA}{THC}$
F30	Street cannabis	0.765	30.0	0.188	0	0	0.0177	0.00406	0.0488	0.00647	0.118	0.0100	0	0	0	0.0518	0
F31	Street cannabis	0.524	33.3	0.0157	0	0	0.0127	0.00968	0	0	0	0	0	0	0	0.0778	0
F32	Street cannabis	0.760	11.2	1.08	0	0	0.0168	0.00332	0.0400	0.0400	0.0880	0.0104	0	0	0	0.380	0
F33	Street cannabis	0	7.32	0.439	0	0	0.0129	0.00976	0	0	0	0	0	0	0	0.317	0
F34	Street cannabis	0.575	7.87	0.0553	0	0	0.0194	0.00936	0	0	0	0	0	0	0	1.72	0
F35	Street cannabis	0.312	16.0	0.0360	0	0	0.0224	0	0.0160	0	0.0440	0	0	0	0	0.208	0
F36	Street cannabis	0.575	21.3	0.0372	0	0	0.0277	0	0	0	0.106	0	0	0	0	0.681	0
F37	Street cannabis	0	5.88	0.0279	0	0	0.0197	0.0121	0	0	0	0	0	0	0	0.353	0
F38	Street cannabis	0.581	20.0	0.0575	0	0	0.0238	0.00325	0.0475	0	0.0625	0	0	0	0.0131	0.263	0
F39	Street cannabis	0.500	9.63	0.0363	0	0	0.0275	0.00625	0	0	0.125	0	0	0	0.0250	0.338	0
F40	Street cannabis	0	10.7	0.0211	0	0	0.0146	0	0	0	0	0	0	0	0	0.571	0
F41	Street cannabis	0.443	7.21	0.0197	0	0	0.0180	0.00984	0.0656	0	0	0	0	0	0	0.180	0
F42	Street cannabis	0.909	2.27	0.0659	0	0	0.00614	0	0	0	0	0	0	0	0	0.152	0
F43	Street cannabis	0.616	23.3	0.00361	0.0547	0.000337	0.0221	0.000605	0.0361	0.00163	0.0233	0.000930	0	0	0.0116	0.0756	0
F44	Street cannabis	0.426	9.79	0.00277	0	0	0.0383	0.0100	0.0851	0	0	0	0	0	0	0.340	0
F45	Street cannabis	0.667	18.3	0.267	0	0	0.0183	0	0	0	0	0	0	0	0	0.317	0
F46	Street cannabis	0.641	4.69	0.344	0	0	0.0188	0	0	0.0145	0	0.0141	0	0	0	0.828	0
F47	Street cannabis	0.300	3.90	0.0210	0	0	0.0240	0.00430	0.0580	0	0	0	0	0	0	0.0460	0
F48	Street cannabis	0.544	8.04	0.0674	0	0	0.0200	0	0	0	0	0	0	0	0	0.239	0
F49	Street cannabis	0.449	7.98	0.0303	0	0	0.0135	0	0	0	0	0	0	0	0	0.393	0
F50	Street cannabis	0	5.00	2.20	0	0	0.00900	0.0850	0	0	0	0.0600	0	0	0	1.00	0
F51	Street cannabis	0	12.0	0.0622	0	0	0.0222	0.00911	0	0	0	0	0	0	0	0.289	0
F52	Street cannabis	0.690	6.90	0.0345	0	0	0.0207	0	0	0	0	0	0	0	0	0.169	0

Table S3. Cont.

Sample	Substance intake	$\frac{THC-OH}{THC}$	$\frac{THC-COOH}{THC}$	$\frac{THCAA}{THC}$	$\frac{CBD}{THC}$	$\frac{CBDA}{THC}$	$\frac{CBN}{THC}$	$\frac{CBNA}{THC}$	$\frac{CBG}{THC}$	$\frac{CBGA}{THC}$	$\frac{CBC}{THC}$	$\frac{CBCA}{THC}$	$\frac{CBL}{THC}$	$\frac{CBLA}{THC}$	$\frac{THCV}{THC}$	$\frac{THCVA}{THC}$	$\frac{CBDVA}{THC}$
F53	Street cannabis	0.541	14.9	0.0541	0	0	0.0595	0	0	0	0	0	0	0	0	0.205	0
F54	Street cannabis	0	5.00	0.650	0	0	0.00650	0	0	0.0480	0	0	0	0	0	0.225	0
F55	Street cannabis	0.536	13.4	0.232	0	0	0.0179	0	0	0.0357	0	0	0	0	0	0.446	0

Samples with THC concentrations <LOD were shaded grey. For other cannabinoid concentrations <LOD, 0 was substituted as a ratio.

Table S4. Cannabinoid serum concentrations of study and forensic serum samples standardized on THC (Cannabinoid ratios)

Sample	Substance intake	$\frac{THC-OH}{THC}$	$\frac{THC-COOH}{THC}$	$\frac{THCAA}{THC}$	$\frac{CBD}{THC}$	$\frac{CBDA}{THC}$	$\frac{CBN}{THC}$	$\frac{CBNA}{THC}$	$\frac{CBG}{THC}$	$\frac{CBGA}{THC}$	$\frac{CBC}{THC}$	$\frac{CBCA}{THC}$	$\frac{CBL}{THC}$	$\frac{CBLA}{THC}$	$\frac{THCV}{THC}$	$\frac{THCVA}{THC}$	$\frac{CBDVA}{THC}$
Sat01	Sativex	0.448	13.8	0.0483	0.135	0.00148	0.0414	0	0.0379	0.00517	0.145	0	0	0	0.0166	0.117	0
Sat02	Sativex	0.508	20.0	0.262	0.231	0.0939	0.0246	0.00339	0.0339	0.00831	0.129	0.0152	0	0	0.00400	0.142	0
Sat03	Sativex	0.711	14.0	0.316	0.0421	0.00368	0.0474	0.00474	0.0421	0.0124	0.121	0.00632	0	0	0.0211	0.158	0
Sat04	Sativex	0.367	4.67	0.0225	0.117	0.000183	0.167	0.00417	0.0383	0.00225	0.150	0.00117	0	0	0.0150	0.0300	0
Dro01	Dronabinol	0.417	31.3	0.0521	0.323	0	0.125	0.0115	0.0500	0	0.375	0	0	0	0	0.302	0
Dro02	Dronabinol	0.231	17.7	0.0354	0	0	0.0408	0	0.0923	0	0.200	0.123	0	0	0	0.200	0
Dro03	Dronabinol	0.407	17.0	0.441	0.0107	0	0.0322	0.00373	0.0593	0.0322	0.288	0.00661	0	0	0.0114	0.492	0
Dro04	Dronabinol	1.58	45.3	0.0842	0	0	0.00790	0	0	0	0	0	0	0	0	0.163	0
THCM01	THC medicine	0.238	4.67	0.0571	0.0410	0	0.0214	0.00667	0.0371	0	0.0905	0.00433	0	0	0	0.414	0

For cannabinoid concentrations <LOD, 0 was substituted as a ratio.

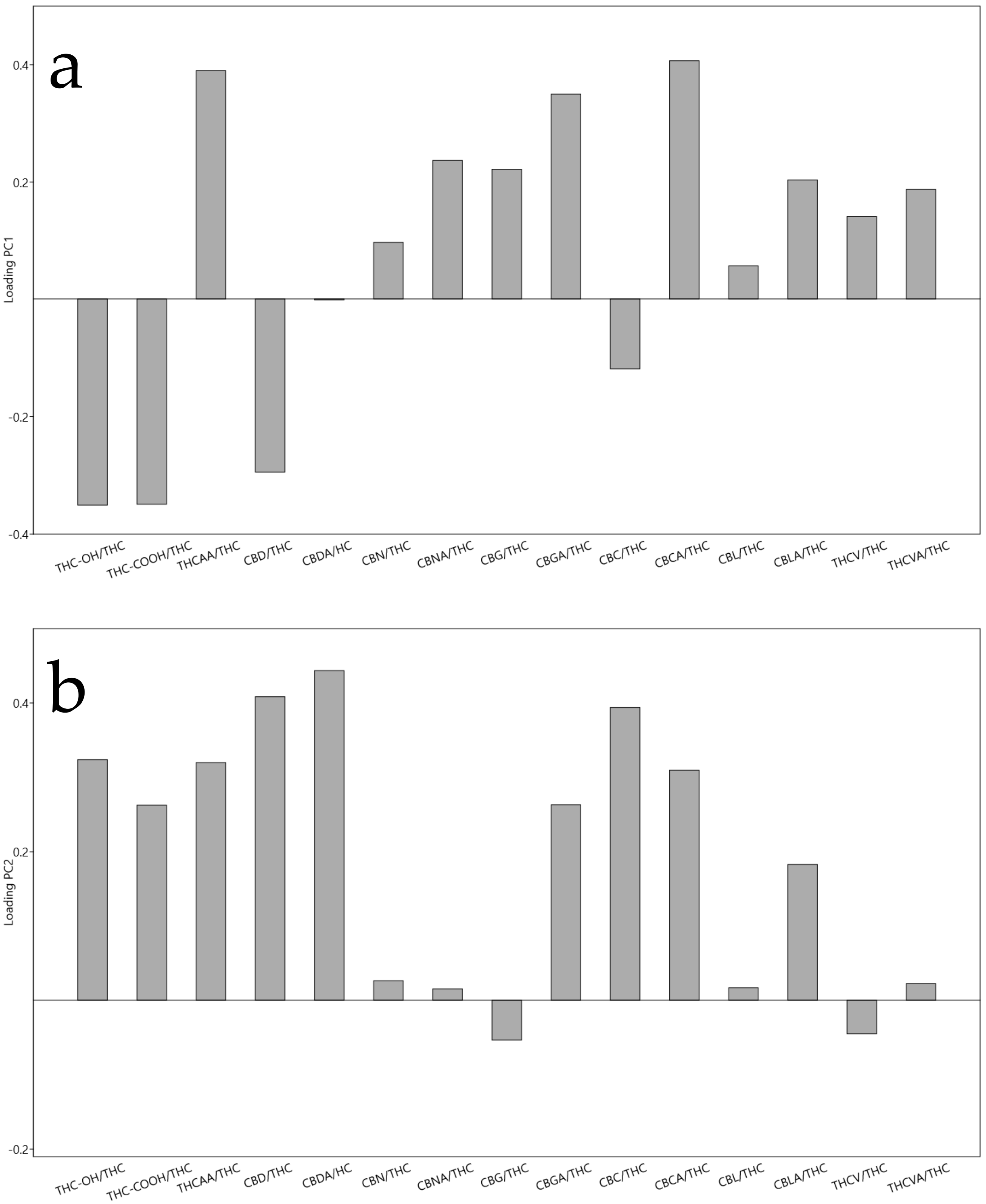

Figure S1. Loading plot of principal component (PC) 1 and PC 2 for PCA of study and forensic samples. PC 1 (**a**) has negative loadings for THC-OH/THC, THC-COOH/THC, CBD/THC and CBC/THC, while especially THCAA/THC, CBGA/THC and CBCA/THC are correlated positively. PC 2 (**b**) has positive loadings for most variables, except for CBG/THC and THCV/THC.

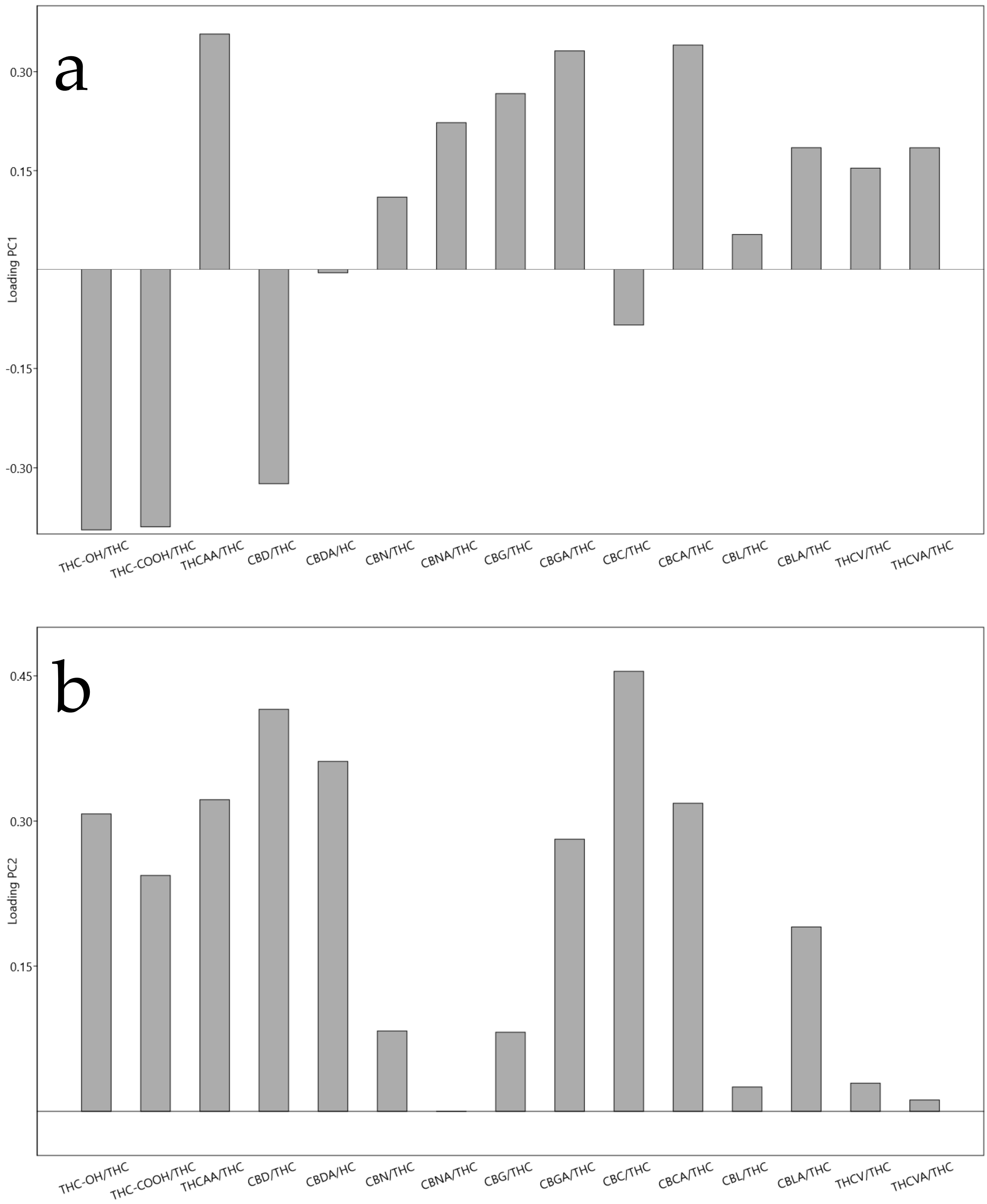

Figure S2. Loading plot of principal component (PC) 1 and PC 2 for PCA of study samples, forensic samples of street cannabis users and forensic samples with reported intake of cannabis-based medicines. Similar to supplementary Figure 1, PC 1 (**a**) has negative loadings for THC-OH/THC, THC-COOH/THC, CBD/THC and CBC/THC, while most other cannabinoids, especially THCAA/THC, CBGA/THC and CBCA/THC, are correlated positively. PC 2 (**b**) has positive loadings for almost all variables.
